# Supplementary material for: Functional constraints of wtf killer meiotic drivers
Source: bioRxiv. 2025 Jan 9:2024.08.27.609905. Preprint. [Version 3] doi: 10.1101/2024.08.27.609905 (PMC11642804; doi:10.1101/2024.08.27.609905)

bioRxiv preprint doi: https://doi.org/10.1101/2024.08.27.609905; this version posted January 9, 2025. The copyright holder for this preprint (which was not certified by peer review) is the author/funder, who has granted bioRxiv a license to display the preprint in perpetuity. It is made available under aCC-BY 4.0 International license.

S1 Figure

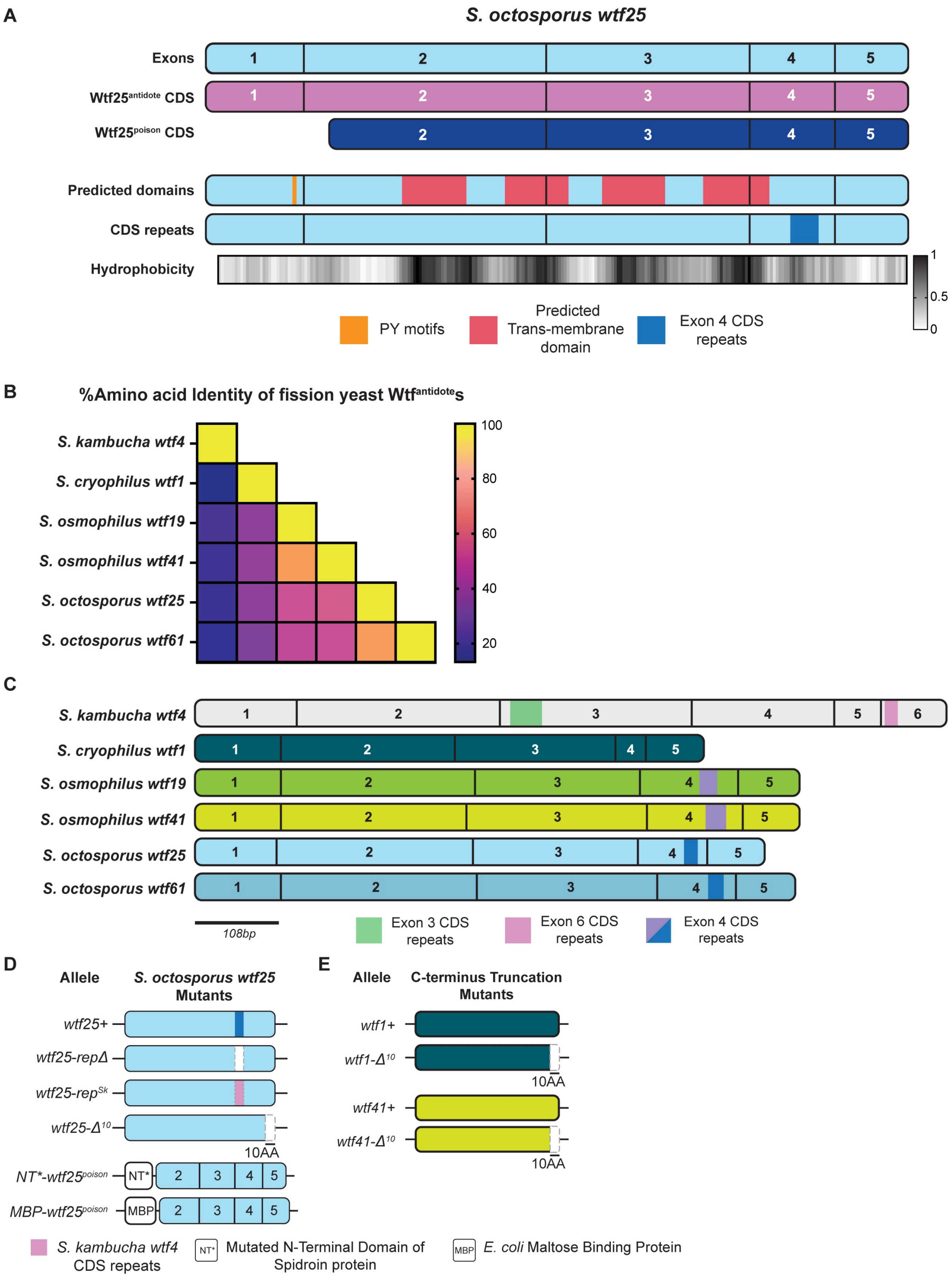

bioRxiv preprint doi: https://doi.org/10.1101/2024.08.27.609905; this version posted January 9, 2025. The copyright holder for this preprint (which was not certified by peer review) is the author/funder, who has granted bioRxiv a license to display the preprint in perpetuity. It is made available under aCC-BY 4.0 International license.

S2 Figure

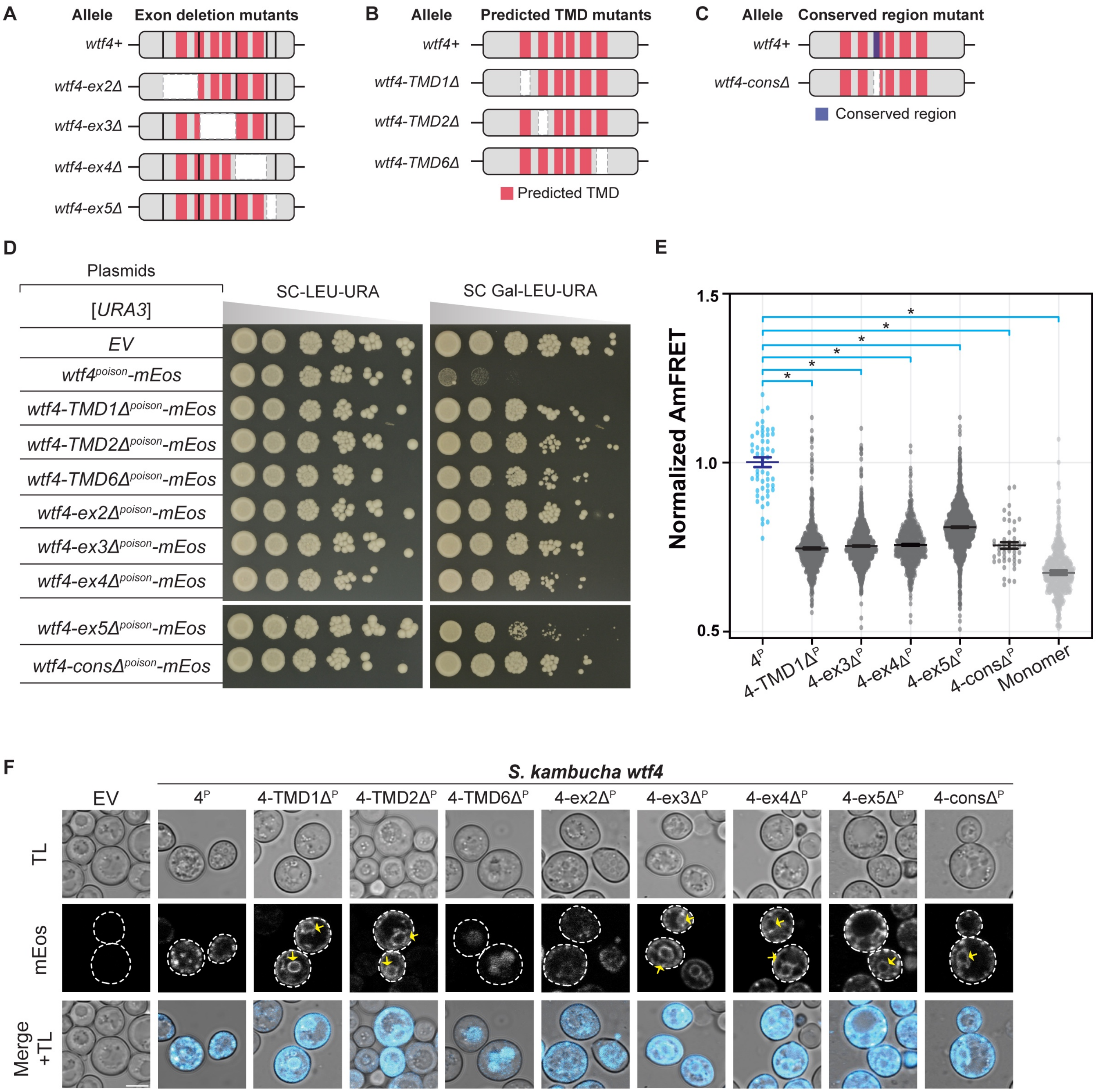

bioRxiv preprint doi: https://doi.org/10.1101/2024.08.27.609905; this version posted January 9, 2025. The copyright holder for this preprint (which was not certified by peer review) is the author/funder, who has granted bioRxiv a license to display the preprint in perpetuity. It is made available under aCC-BY 4.0 International license.

S3 Figure

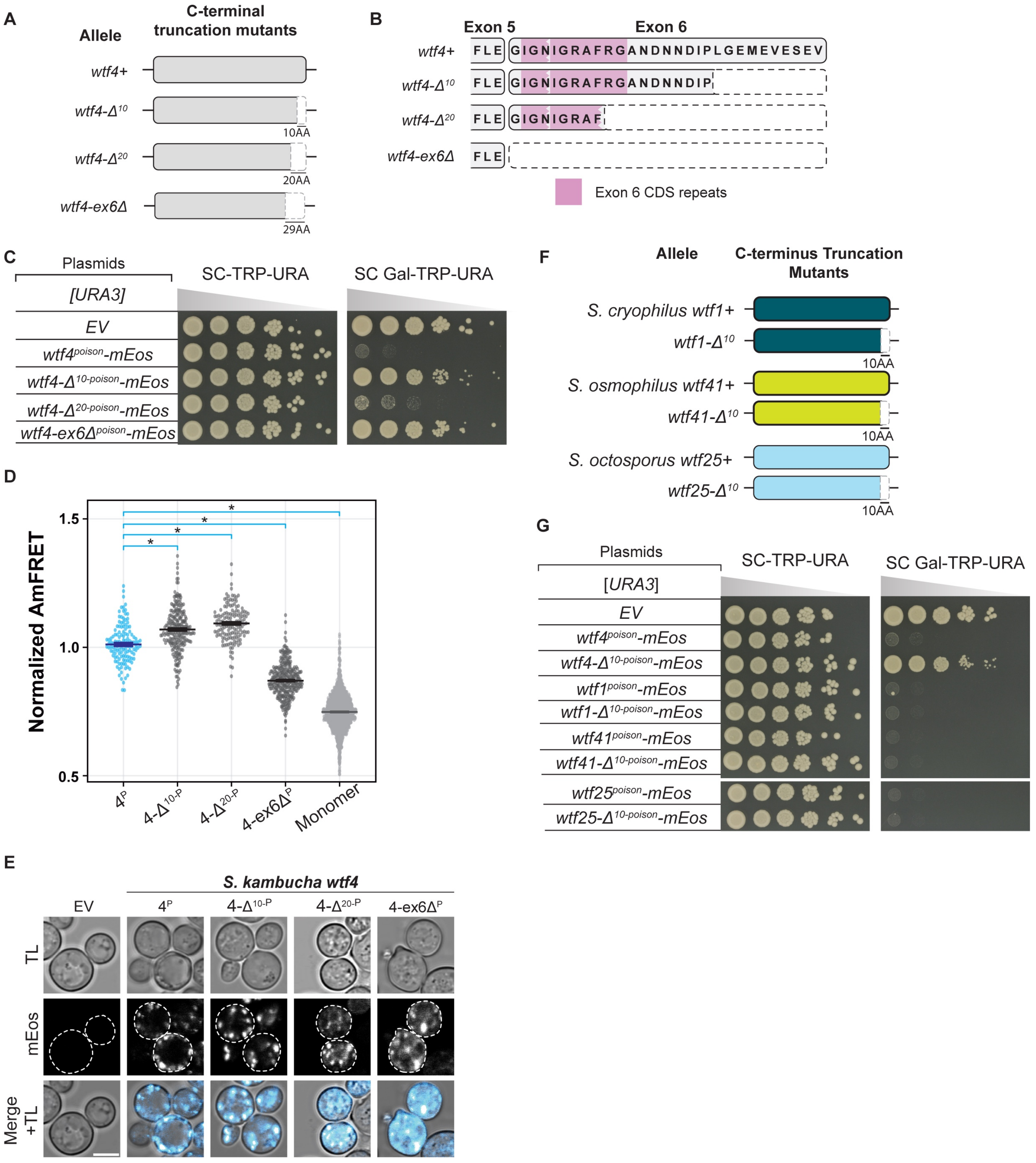

bioRxiv preprint doi: https://doi.org/10.1101/2024.08.27.609905; this version posted January 9, 2025. The copyright holder for this preprint (which was not certified by peer review) is the author/funder, who has granted bioRxiv a license to display the preprint in perpetuity. It is made available under aCC-BY 4.0 International license.

S4 Figure

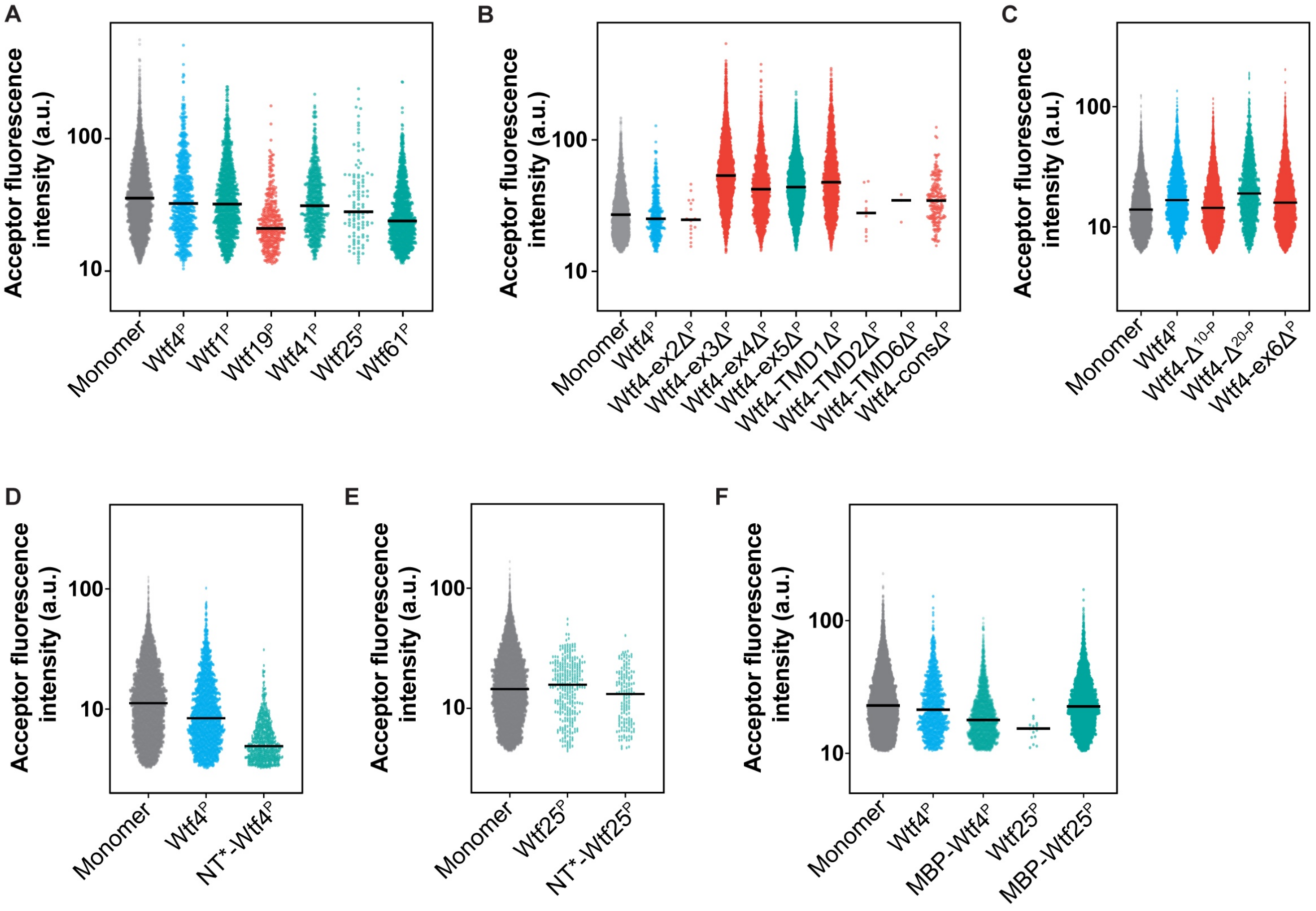

bioRxiv preprint doi: https://doi.org/10.1101/2024.08.27.609905; this version posted January 9, 2025. The copyright holder for this preprint (which was not certified by peer review) is the author/funder, who has granted bioRxiv a license to display the preprint in perpetuity. It is made available under aCC-BY 4.0 International license.

S5 Figure

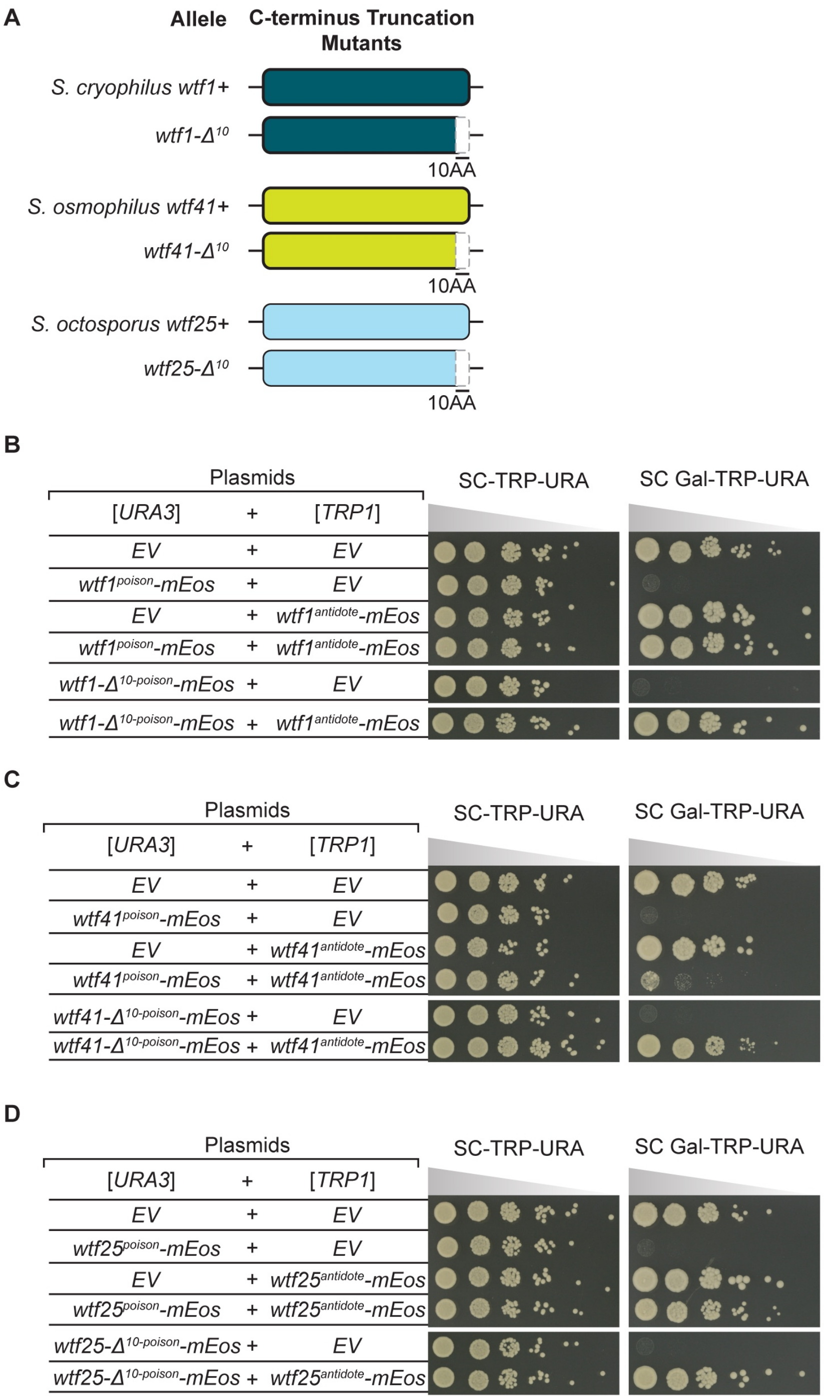

bioRxiv preprint doi: https://doi.org/10.1101/2024.08.27.609905; this version posted January 9, 2025. The copyright holder for this preprint (which was not certified by peer review) is the author/funder, who has granted bioRxiv a license to display the preprint in perpetuity. It is made available under aCC-BY 4.0 International license.

S6 Figure

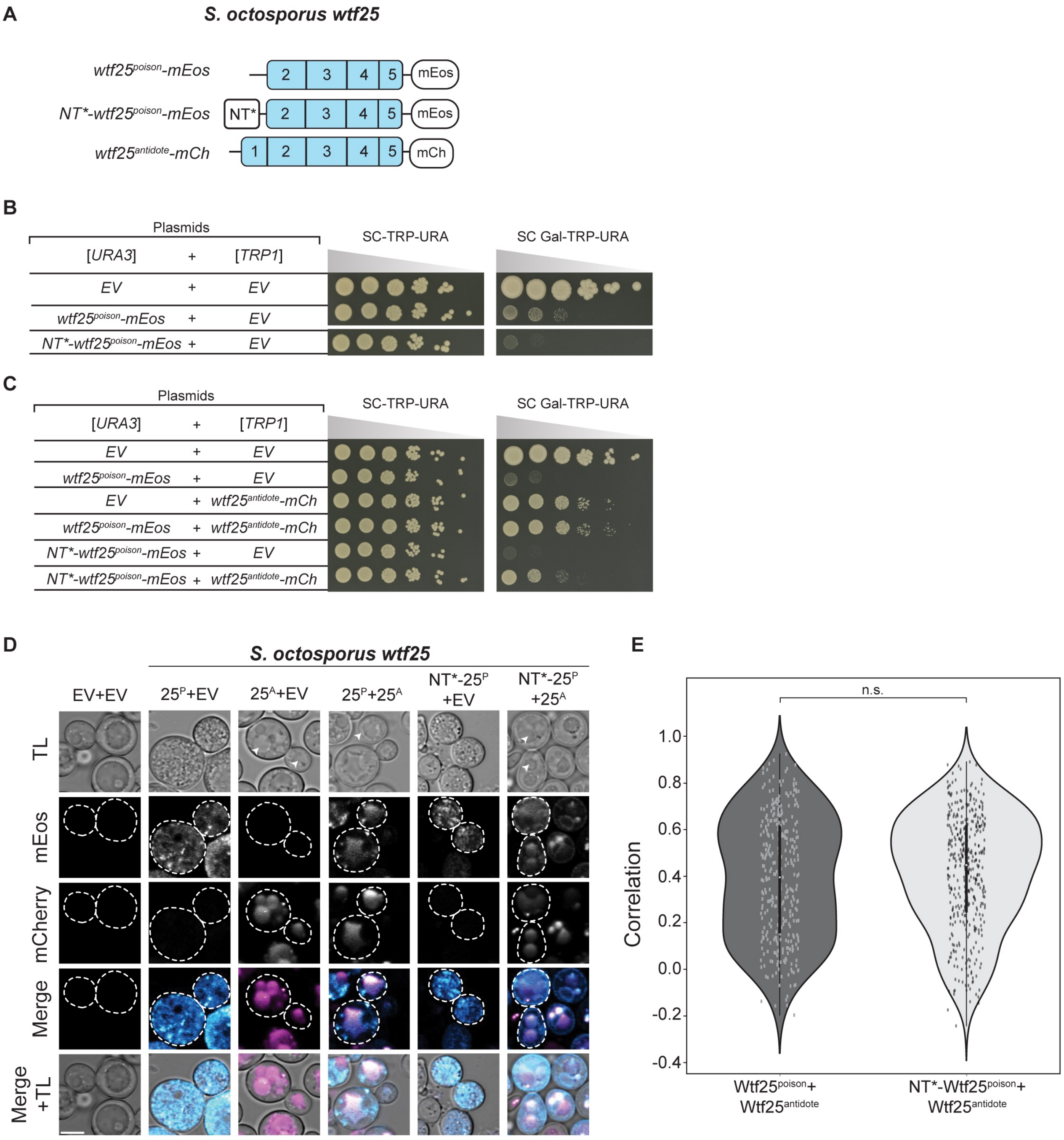

bioRxiv preprint doi: https://doi.org/10.1101/2024.08.27.609905; this version posted January 9, 2025. The copyright holder for this preprint (which was not certified by peer review) is the author/funder, who has granted bioRxiv a license to display the preprint in perpetuity. It is made available under aCC-BY 4.0 International license.

S7 Figure

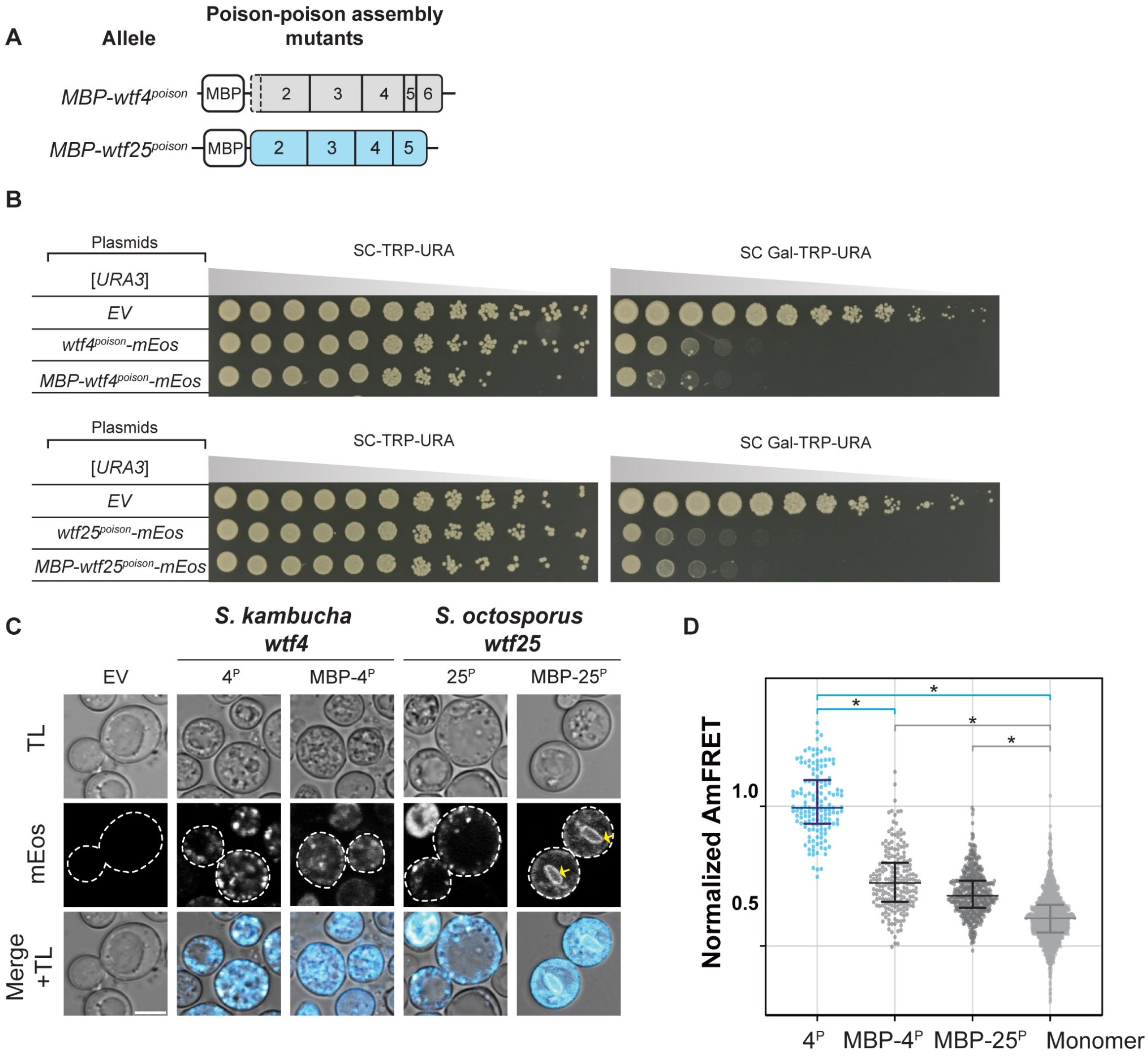

bioRxiv preprint doi: https://doi.org/10.1101/2024.08.27.609905; this version posted January 9, 2025. The copyright holder for this preprint (which was not certified by peer review) is the author/funder, who has granted bioRxiv a license to display the preprint in perpetuity. It is made available under aCC-BY 4.0 International license.

S8 Figure

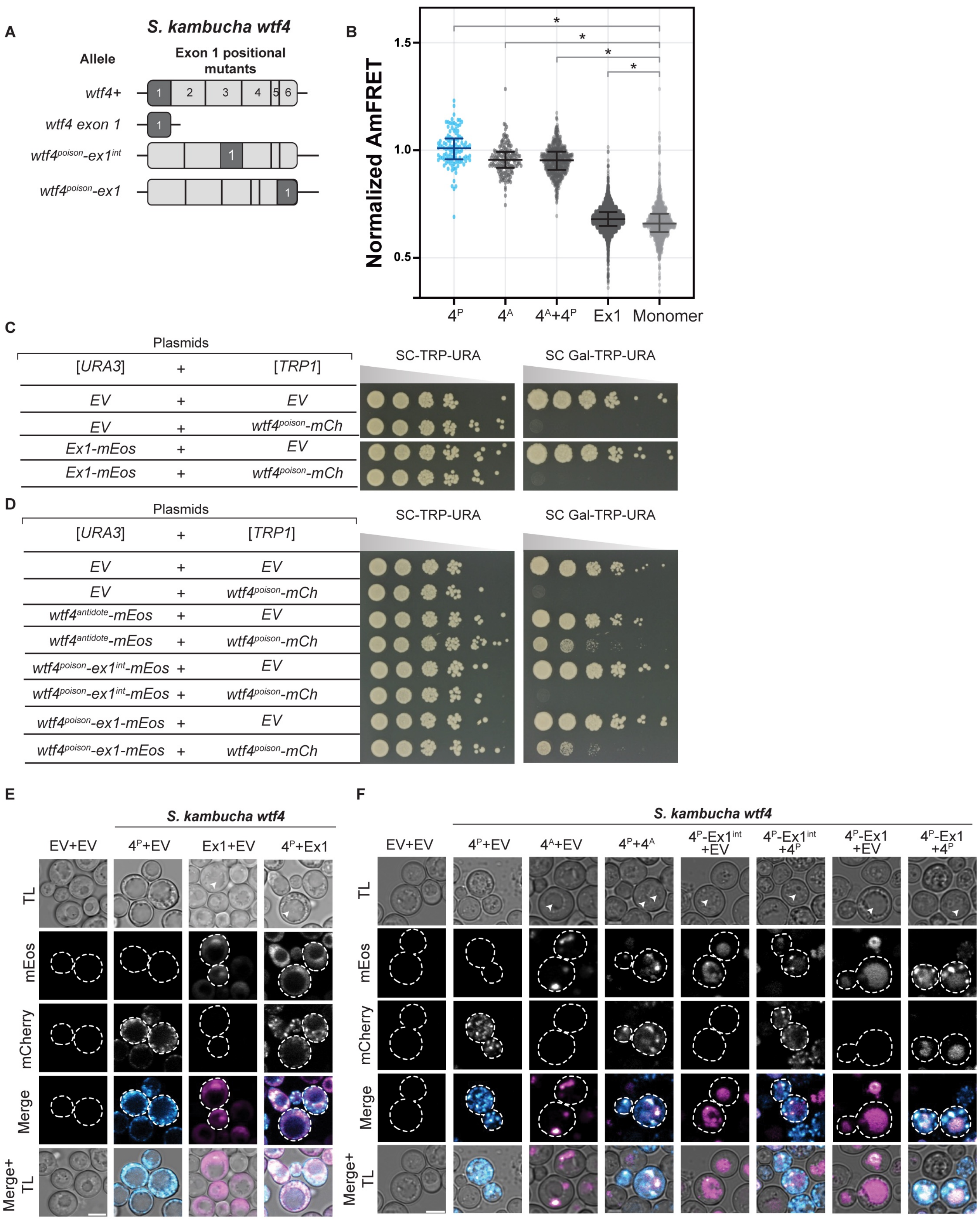

bioRxiv preprint doi: https://doi.org/10.1101/2024.08.27.609905; this version posted January 9, 2025. The copyright holder for this preprint (which was not certified by peer review) is the author/funder, who has granted bioRxiv a license to display the preprint in perpetuity. It is made available under aCC-BY 4.0 International license.

S9 Figure

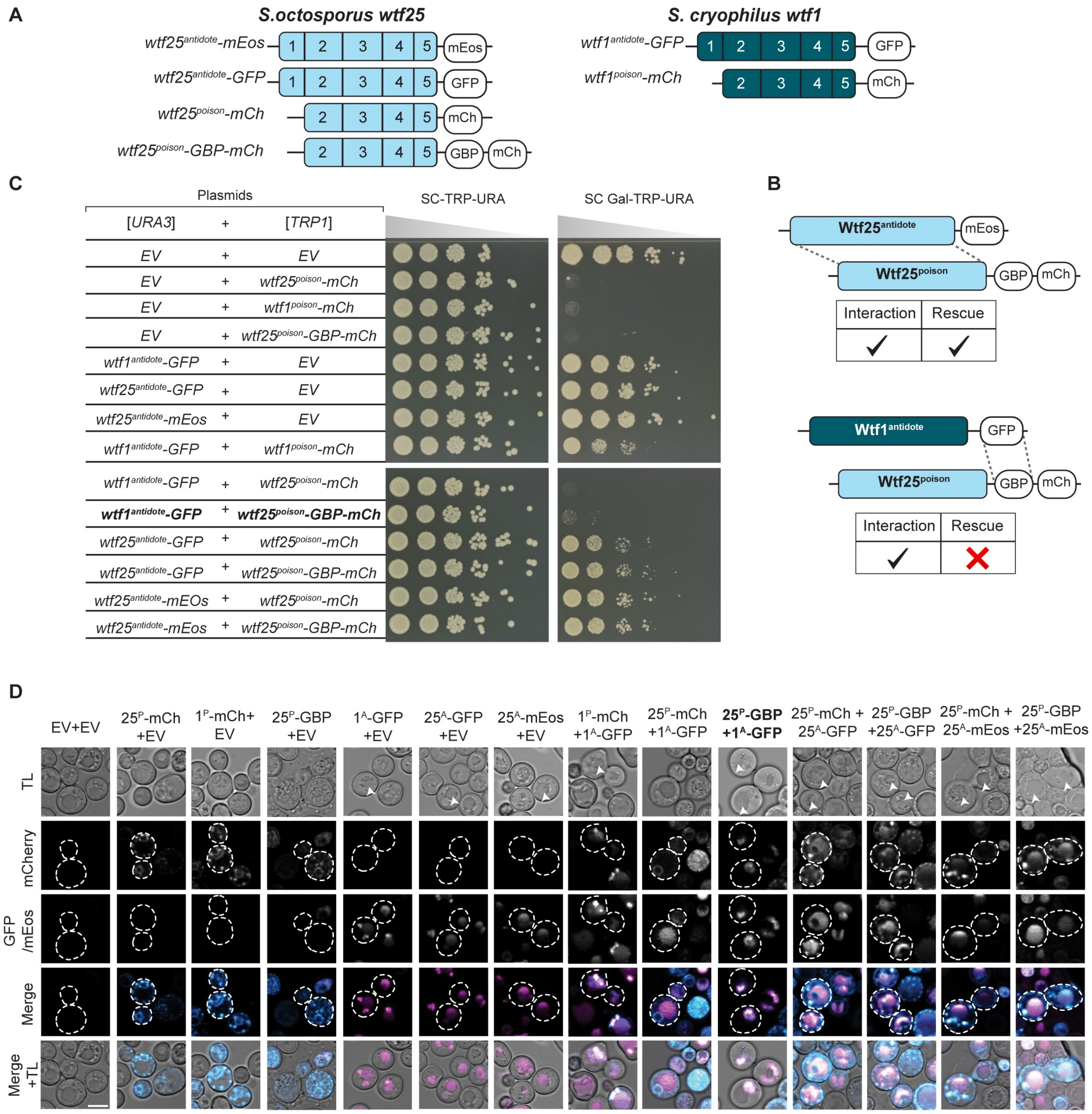

bioRxiv preprint doi: https://doi.org/10.1101/2024.08.27.609905; this version posted January 9, 2025. The copyright holder for this preprint (which was not certified by peer review) is the author/funder, who has granted bioRxiv a license to display the preprint in perpetuity. It is made available under aCC-BY 4.0 International license.

S10 Figure

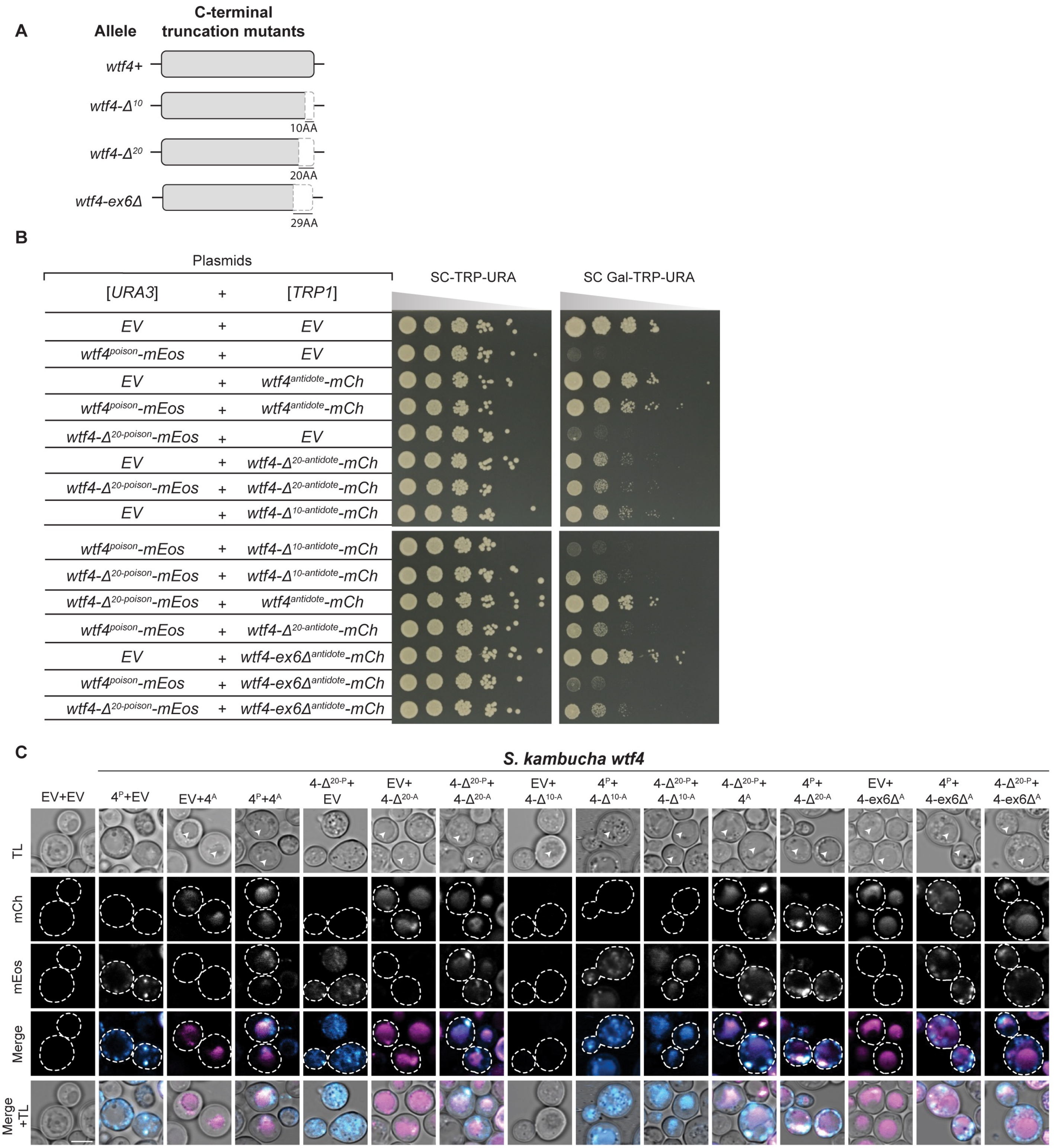

bioRxiv preprint doi: https://doi.org/10.1101/2024.08.27.609905; this version posted January 9, 2025. The copyright holder for this preprint (which was not certified by peer review) is the author/funder, who has granted bioRxiv a license to display the preprint in perpetuity. It is made available under aCC-BY 4.0 International license.

S11 Figure

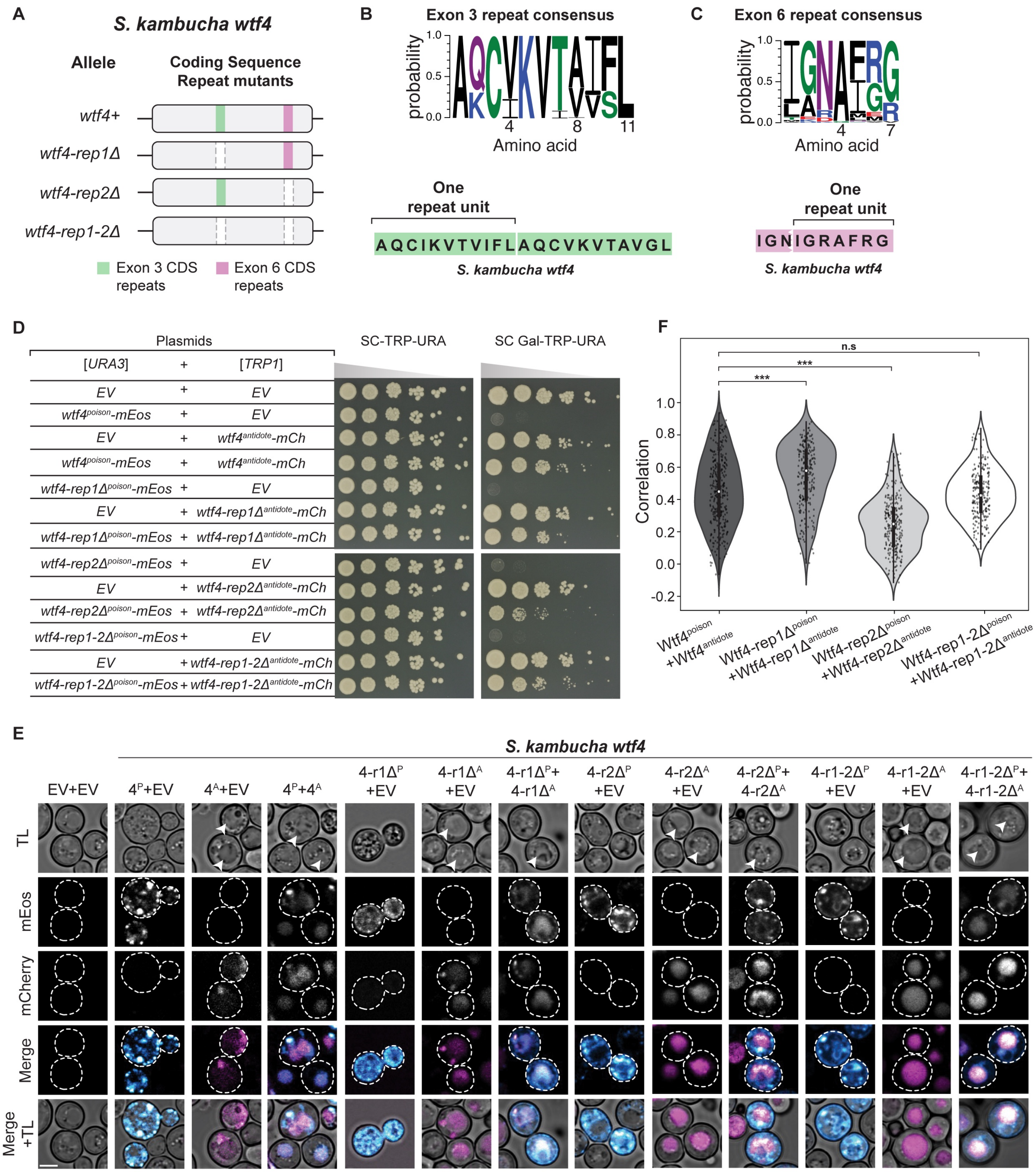

bioRxiv preprint doi: https://doi.org/10.1101/2024.08.27.609905; this version posted January 9, 2025. The copyright holder for this preprint (which was not certified by peer review) is the author/funder, who has granted bioRxiv a license to display the preprint in perpetuity. It is made available under aCC-BY 4.0 International license.

S12 Figure

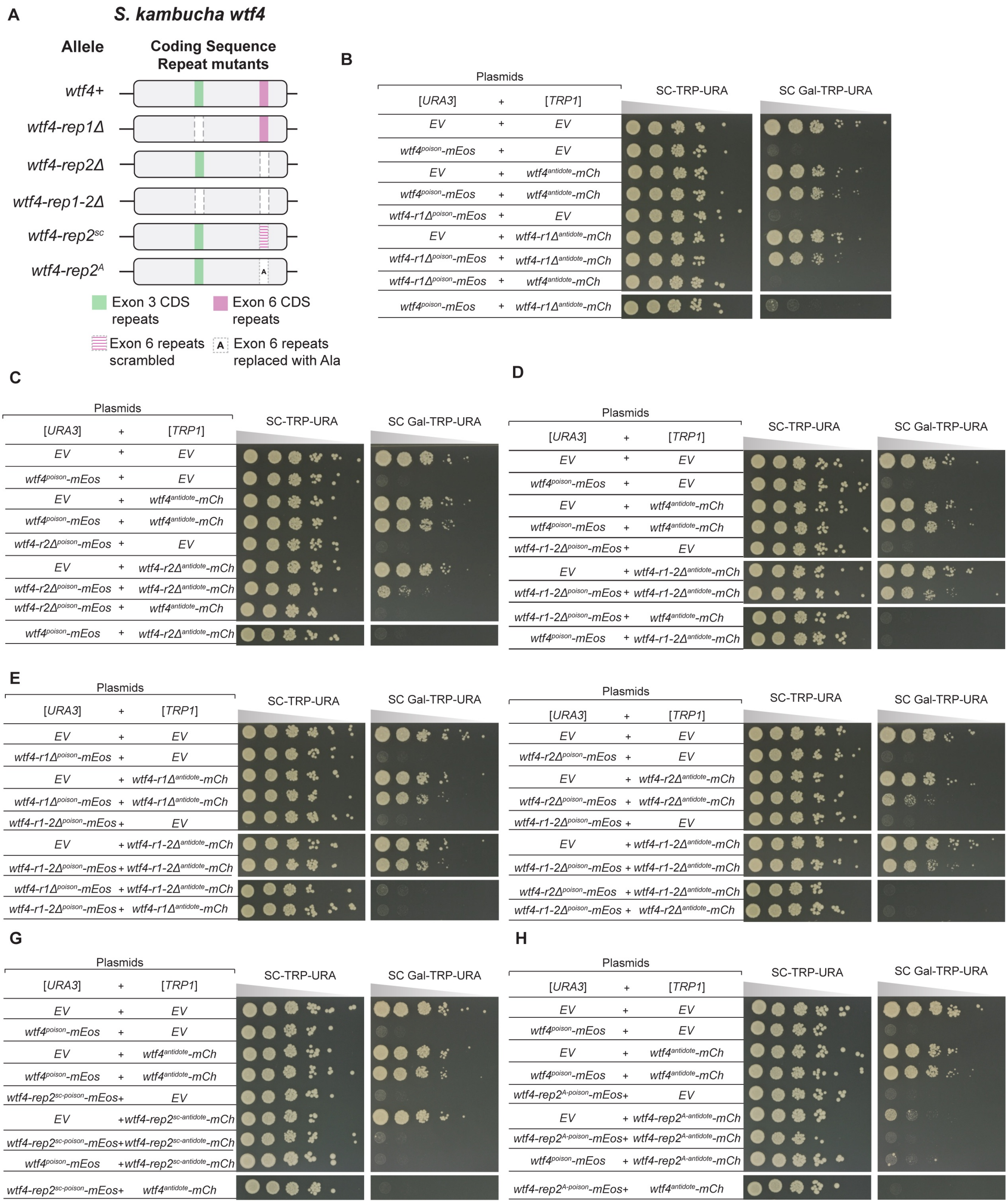

bioRxiv preprint doi: https://doi.org/10.1101/2024.08.27.609905; this version posted January 9, 2025. The copyright holder for this preprint (which was not certified by peer review) is the author/funder, who has granted bioRxiv a license to display the preprint in perpetuity. It is made available under aCC-BY 4.0 International license.

S13 Figure

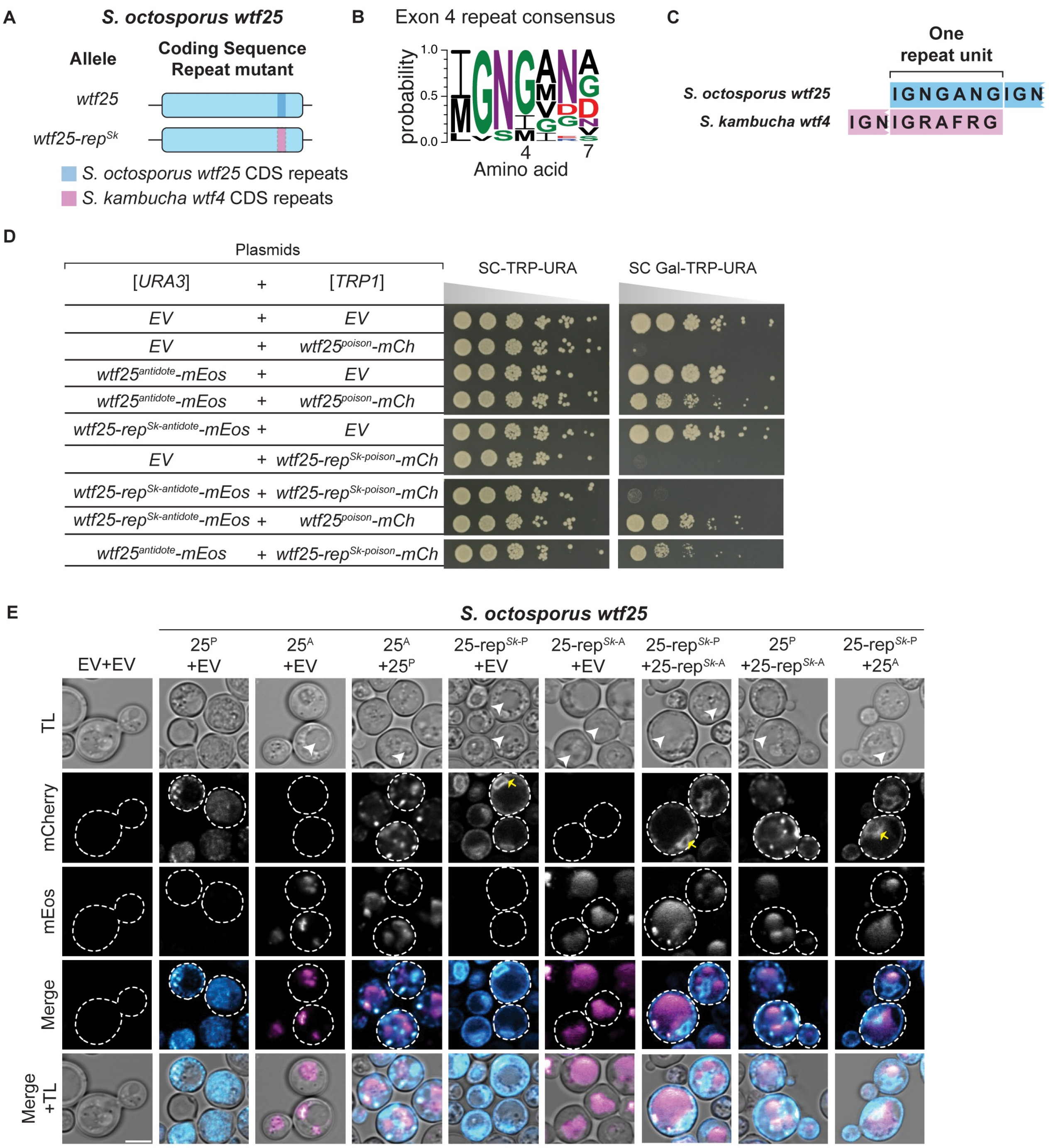

bioRxiv preprint doi: https://doi.org/10.1101/2024.08.27.609905; this version posted January 9, 2025. The copyright holder for this preprint (which was not certified by peer review) is the author/funder, who has granted bioRxiv a license to display the preprint in perpetuity. It is made available under aCC-BY 4.0 International license.

S14 Figure

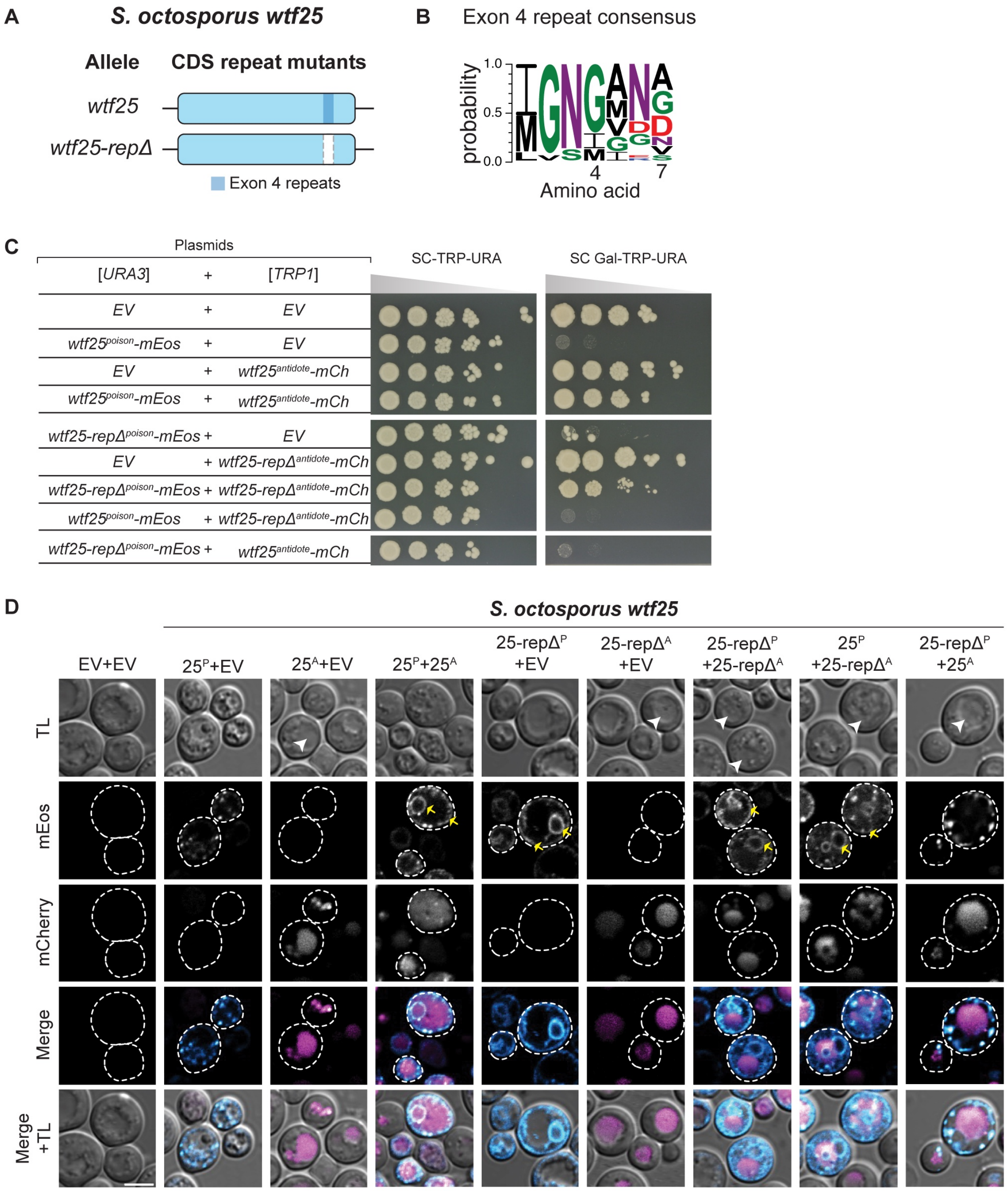

Supplement: 1 — S1 Figure. Wtf proteins share limited amino acid identity but have common features A. A cartoon of S. octosporus wtf25 coding sequence (CDS). Wtf25antidote coding sequence is shown in purple, which includes exons 1–5. The Wtf4poison coding sequence is shown in navy, which begins at the 27th base pair of exon 2 and extends through exon 5. Row 4 depicts the predicted transmembrane domains (in red) and PY motif (in mustard). Row 5 depicts the CDS repeats found in exon 4 (in cobalt). Row 6 depicts the normalized hydrophobicity of Wtf25 proteins from ProtScale, with the Kyle and Doolittle Hydropathy scale [81]. The higher the number on the scale, the higher the hydrophobicity of the amino acid. See S2 and S3 Tables for more detailed descriptions. B. Pairwise amino acid identity of the 6 Wtfantidote proteins shown. The amino acid sequences were aligned using Geneious Prime (2023.0.4), and the percentage amino acid identity is depicted as a heatmap, with yellow being 100% identity. C. Depiction of CDS repeats and lengths of 6 wtfantidote CDSs. The CDS repeats found in exon 6 of S. kambucha wtf4 are homologous to those found in exon 4 of the other wtf genes [19]. The scale bar represents 108 base pairs (bp). D-E. S. octosporus wtf25 (D), S. cryophilus wtf1, and S. osmophilus wtf41 (E) mutants constructed in this study. The categories have their respective wild type allele shown on top. See S1 Table for a comprehensive overview of the alleles and their phenotypes. S2 Figure. Deletion mutants affect Wtf4poison toxicity, self-assembly and localization. Cartoon of S. kambucha wtf4 exon deletion mutants (A), predicted transmembrane domain (TMD) deletion mutants (B) and a mutant that deletes a 9 amino acid conserved region encoded in exon 3 (C). D. A spot assay of cells serially diluted and plated on SC-LEU-URA and SC Gal-LEU-URA plates and grown at 30°C for 3 days. Each strain carries an empty [LEU2] plasmid, and either an empty [URA3] plasmid (EV) or the indicated wtf4poison-mE [file NIHPP2024.08.27.609905V3-supplement-1.pdf]
