# Supplementary material for: Functional constraints of wtf killer meiotic drivers
Source: bioRxiv. 2024 Dec 2:2024.08.27.609905. Preprint. [Version 2] doi: 10.1101/2024.08.27.609905 (PMC11642804; doi:10.1101/2024.08.27.609905)
Supplement: Supplementary file 1 [file NIHPP2024.08.27.609905v2-supplement-9.pdf]

bioRxiv preprint doi: https://doi.org/10.1101/2024.08.27.609905; this version posted December 2, 2024. The copyright holder for this preprint (which was not certified by peer review) is the author/funder, who has granted bioRxiv a license to display the preprint in perpetuity. It is made available under aCC-BY 4.0 International license.

Supplemental Information

S1 Figure. Wtf proteins share limited amino acid identity but have common features

A. A cartoon of *S. octosporus wtf25* coding sequence (CDS). Wtf25^{antidote} coding sequence is shown in purple, which includes exons 1-5. The Wtf4^{poison} coding sequence is shown in navy, which begins at the 27th base pair of exon 2 and extends through exon 5. Row 4 depicts the predicted transmembrane domains (in red) and PY motif (in mustard). Row 5 depicts the CDS

bioRxiv preprint doi: https://doi.org/10.1101/2024.08.27.609905; this version posted December 2, 2024. The copyright holder for this preprint (which was not certified by peer review) is the author/funder, who has granted bioRxiv a license to display the preprint in perpetuity. It is made available under aCC-BY 4.0 International license.

repeats found in exon 4 (in cobalt). Row 6 depicts the normalized hydrophobicity of Wtf25 proteins from ProtScale, with the Kyle and Doolittle Hydropathy scale [80]. The higher the number on the scale, the higher the hydrophobicity of the amino acid. See S5 and S6 Tables for more detailed descriptions. **B.** Pairwise amino acid identity of the 6 Wtf^{antidote} proteins shown. The amino acid sequences were aligned using Geneious Prime (2023.0.4), and the percentage amino acid identity is depicted as a heatmap, with yellow being 100% identity. **C.** Depiction of CDS repeats and lengths of 6 *wtf*^{antidote} CDSs. The CDS repeats found in exon 6 of *S. kambucha wtf4* are homologous to those found in exon 4 of the other *wtf* genes [19]. The scale bar represents 108 base pairs (bp). **D-E.** *S. octosporus wtf25* (**D**), *S. cryophilus wtf1*, and *S. osmophilus wtf41* (**E**) mutants constructed in this study. The categories have their respective wild type allele shown on top. See S1 Table for a comprehensive overview of the alleles and their phenotypes.

S2 Figure. Deletion mutants affect Wtf4^{poison} toxicity, self-assembly and localization. Cartoon of *S. kambucha wtf4* exon deletion mutants (**A**), predicted transmembrane domain (TMD) deletion mutants (**B**) and a mutant that deletes a 9 amino acid conserved region encoded in exon 3 (**C**). **D.** A spot assay of cells serially diluted and plated on SC-LEU-URA and SC Gal-LEU-URA plates and grown at 30•C for 3 days. Each strain carries an empty [*LEU2*] plasmid, and either an empty [*URA3*] plasmid (EV) or the indicated *wtf4*^{poison}*-mEos* alleles under the control of a galactose-inducible promoter. The horizontal break in the image for each plate is due to rearrangement of the image to facilitate easy comparison. All strains were grown on the same plates (i.e., one SC-LEU-URA or SC Gal-LEU-URA plate). **E.** AmFRET values for three technical replicates of the specified Wtf4^{poison}-mEos proteins and monomer-mEos (negative control). The median is indicated with a solid line and the bars represent the interquartile range. For easier comparison, the values were normalized so that Wtf4^{poison} had a median of 1 in each experiment. The data shown here does not include outliers. See S2 Data for the complete dataset and p-values. Statistical significance: *p<0.008, **p<0.0008, t-tests between the means of each replicate, with Bonferroni correction. **F.** Representative images of the same strains depicted in D were induced in galactose media for 4 hours at 30•C to express the indicated mEos-tagged proteins. The images are not at the same brightness and contrast settings to clearly show localization of tagged proteins. Yellow arrows indicate endoplasmic reticulum-like localization. 4^{P} indicates Wtf4^{poison}, TL is transmitted light, and the scale bar is 4 µm.

S3 Figure. C-terminal truncations have inconsistent effects on Wtf^{poison} protein toxicity.

bioRxiv preprint doi: https://doi.org/10.1101/2024.08.27.609905; this version posted December 2, 2024. The copyright holder for this preprint (which was not certified by peer review) is the author/funder, who has granted bioRxiv a license to display the preprint in perpetuity. It is made available under aCC-BY 4.0 International license.

A. Cartoon of C-terminal truncation mutants of *S. kambucha wtf4*. **B.** Cartoon illustrating the amino acids lost with the C-terminal truncation alleles of *wtf4*. The amino acids comprising the exon 6 coding sequence repeats are highlighted in pink. **C.** A spot assay of cells serially diluted and plated on SC-TRP-URA and SC Gal-TRP-URA plates and grown at 30•C for 3 days. Each strain carries both an empty [*TRP1*] plasmid (EV) and a [*URA3*] plasmid that is either empty or carries the indicated $wtf4^{poison}$*-mEos* allele under the control of a galactose inducible promoter. **D.** AmFRET values for three technical replicates of the specified $Wtf4^{poison}$-mEos alleles and monomer-mEos (negative control). The median is indicated with a solid line and the bars represent the interquartile range. For easier comparison, the values were normalized so that $Wtf4^{poison}$ had a median of 1 in each experiment. The data shown here does not include outliers. See S2 Data for the complete dataset and p-values. Statistical significance: *p<0.0125, t-test with Bonferroni correction. **E.** Representative images of the same strains depicted in C were induced with galactose media for 4 hours at 30•C to express the indicated mEos-tagged proteins. The images are not at the same brightness and contrast settings to clearly show localization of tagged proteins. 4^{P} indicates $Wtf4^{poison}$, TL is transmitted light, and the scale bar is 4 µm. **F.** Cartoons of C-terminal truncation mutants of *S. cryophilus wtf1* (*wtf1-*Δ^{10}), *S. osmophilus wtf41* (*wtf41-*Δ^{10}), and *S. octosporus wtf25* (*wtf25-*Δ^{10}). **G.** Spot assays of cells serially diluted and plated on SC-URA and SC Gal-URA plates and grown at 30•C for 3 days. Each strain carries both an empty [*TRP1*] plasmid (EV) and an empty [*URA3*] plasmid (EV), or the indicated wtf^{poison}*-mEos* allele under the control of a galactose-inducible promoter. The horizontal breaks in the images for each plate are due to rearrangements of the image to facilitate easy comparison. All strains were grown on the same plates (i.e., one SC-TRP-URA or SC Gal-TRP-URA plate).

S4 Figure. Reducing assembly with NT* tag increases $Wtf25^{poison}$ toxicity.

A. Cartoon of alleles used in this experiment (B-E). The NT* tag has a general anti-aggregation property [33]. **B-C.** Spot assays of cells serially diluted on SC-TRP-URA and SC Gal-TRP-URA plates and grown at 30•C for 4 days. Each strain carries both a [*URA3*] and a [*TRP1*] plasmid. The plasmids are either empty (EV) or carry the indicated *wtf25* alleles under the control of galactose-inducible promoters. In B, the horizontal break in the image of each plate is due to a rearrangement of the image to facilitate easy comparison. All strains within a panel were grown on the same plates (i.e., one SC-TRP-URA or SC Gal-TRP-URA plate for panel B). **D.** Representative images of the same strains shown in C were induced with galactose media for 4 hours at 30•C to express the indicated $Wtf25^{poison}$-mEos proteins and/or $Wtf25^{antidote}$-mCherry.

bioRxiv preprint doi: https://doi.org/10.1101/2024.08.27.609905; this version posted December 2, 2024. The copyright holder for this preprint (which was not certified by peer review) is the author/funder, who has granted bioRxiv a license to display the preprint in perpetuity. It is made available under aCC-BY 4.0 International license.

The images are not at the same brightness and contrast settings to clearly show localization of tagged proteins. 25^{P} indicates $Wtf25^{poison}$, 25^{A} indicates $Wtf25^{antidote}$, TL is transmitted light, and the scale bar is 4 µm. **E**. Pearson's Correlation between mCherry and mEos signal in cells from D expressing the specified proteins. N>100, p>0.05, t-test.

S5 Figure. Reducing Wtf^{poison} assembly with MBP tag affects toxicity.

A. Cartoon of alleles used in this experiment (B-C). MBP is the *E. coli* Maltose Binding Protein [75]. **B.** Spot assay of cells serially diluted and plated on SC-TRP-URA and SC Gal-TRP-URA plates and grown at 30•C for 4 days. Each strain carries both an empty [*TRP1*] plasmid and a [*URA3*] plasmid that is either empty (EV) or carries the indicated *wtf^{poison}-mEos* allele under the control of a galactose-inducible promoter. The horizontal break in the image of each plate is due to rearrangement of the image to facilitate easy comparison. All strains were grown on the same plates (i.e., one SC Gal-TRP-URA plate or SC-Trp-Ura). **C.** Representative images of the same strains depicted in B induced with galactose media for 4 hours at 30•C to express the indicated mEos-tagged proteins. The images are not at the same brightness and contrast settings to clearly show localization of tagged proteins. Yellow arrows indicate endoplasmic reticulum-like localization. 4^{P} indicates $Wtf4^{poison}$, 25^{P} indicates $Wtf25^{poison}$, TL is transmitted light, and the scale bar is 4 µm. **D.** AmFRET values for three technical replicates of the specified Wtf^{poison}-mEos alleles and monomer-mEos (negative control). The median is indicated with a solid line and the bars represent the interquartile range. For easier comparison, the values were normalized so that $Wtf4^{poison}$ had a median of 1 in each experiment. The data shown does not include outliers. See S2 Data for the complete dataset and p-values. Statistical significance: *p<0.016, t-test with Bonferroni correction.

S6 Figure. The fluorescence signal intensity of expressed Wtf^{poison} proteins does not correlate with toxicity.

A-F Acceptor fluorescence intensity (in arbitrary units, a.u.) of similarly sized live cells across all Wtf DAmFRET experiments in this study, with the line indicating the median of the population. Data represented here is from the following experiments: A corresponds to Fig 2B, B corresponds to S2 Fig E, C corresponds to S3 Fig D, D corresponds to Fig 4C, E corresponds alleles in S4 Fig, and F corresponds to S5 Fig D. The Y-axis is scaled to a log10 scale. The data are color coded, with toxic poisons in teal, and non-toxic alleles in orange.

S7 Figure. Limited modularity of the $Wtf4^{antidote}$-specific domain.

bioRxiv preprint doi: https://doi.org/10.1101/2024.08.27.609905; this version posted December 2, 2024. The copyright holder for this preprint (which was not certified by peer review) is the author/funder, who has granted bioRxiv a license to display the preprint in perpetuity. It is made available under aCC-BY 4.0 International license.

A. Cartoon of *wtf4^antidote^* exon 1 and mutants that relocate, or both relocate and mutate the exon. **B.** AmFRET values for three technical replicates of the specified mEos-tagged proteins and monomer-mEos (negative control). The median is indicated with a solid line and the bars represent 1.5 times the interquartile range. For easier comparison, the values were normalized so that $Wtf4^{poison}$ had a median of 1 in each experiment. The data shown does not include outliers. See S2 Data for the complete dataset and p-values. Statistical significance: *$p<0.0125$, t-tests with Bonferroni correction. **C-D.** Spot assays of cells serially diluted on SC-TRP-URA and SC Gal-TRP-URA plates and grown at 30°C for 3 days. Each strain carries both a [*URA3*] and a [*TRP1*] plasmid. The plasmids are either empty (EV) or carry the indicated *wtf4* alleles under the control of galactose-inducible promoters. The horizontal breaks in the images of each plate in panels C and E are due to rearrangements of the images to facilitate easy comparison. All strains within a panel were grown on the same plates (i.e., one SC-TRP-URA or SC Gal-TRP-URA plate for panel C). **E.** Representative images of the same strains shown in C induced with galactose media for 4 hours at 30°C to express the $Wtf4^{poison}$-mEos, Wtf4 exon1-mCherry, or both proteins. **F.** Representative images of the same strains shown in D were induced with galactose media for 4 hours at 30°C to the indicated *wtf4* alleles. In E-F, arrows in the transmitted light images indicate vacuoles. The images are not at the same brightness and contrast settings to clearly show localization of the tagged proteins. 4^P indicates $Wtf4^{poison}$, 4^A indicates $Wtf4^{antidote}$, Ex1 is Wtf4 exon1, TL is transmitted light, and the scale bar is 4 µm.

S8 Figure. Effective neutralization of $Wtf25^{poison}$ requires more than a physical connection to a $Wtf^{antidote}$.

A. Cartoon of constructs used in this experiment. *S. octosporus* $Wtf25^{poison}$ was tagged at the C-terminus with either mCherry or GBP-mCherry (GBP: GFP-binding protein). *S. octosporus* $Wtf25^{antidote}$ was tagged with mEos or GFP. *S. cryophilus* $Wtf1^{antidote}$ was tagged with GFP. *S. cryophilus* $Wtf1^{poison}$ was tagged with mCherry. **B.** Experimental set up and summary of the results shown in C and D. In a matching Wtf protein pair (top), poison-antidote interaction and rescue of poison toxicity is observed. In the mismatched pair (bottom), interaction between GFP and GBP results in a forced interaction between the poison and the antidote (shown in D). This interaction is insufficient to rescue the mismatched poison (shown in C). **C.** Spot assay of cells serially diluted and plated on SC-TRP-URA and SC Gal-TRP-URA plates and grown at 30°C for 3 days. Each strain carries both a [*URA3*] and a [*TRP1*] plasmid. The plasmids are either empty (EV) or carry the indicated *wtf* alleles under the control of galactose-inducible promoters. The horizontal breaks in the images of each plate are due to rearrangements of the image to

bioRxiv preprint doi: https://doi.org/10.1101/2024.08.27.609905; this version posted December 2, 2024. The copyright holder for this preprint (which was not certified by peer review) is the author/funder, who has granted bioRxiv a license to display the preprint in perpetuity. It is made available under aCC-BY 4.0 International license.

facilitate easy comparison. All strains within a panel were grown on the same plates (i.e., one SC-TRP-URA or SC Gal-TRP-URA plate). **D.** Representative images the strains depicted in C were induced in galactose for 4 hours at 30•C to express the indicated Wtf proteins. The images are not at the same brightness and contrast settings to clearly show localization of tagged proteins. 25^{P} indicates Wtf25^{poison}, 25^{A}- indicates Wtf25^{antidote}, 1^{P} indicates Wtf1^{poison}, 1^{A} indicates Wtf1^{antidote}, TL indicates transmitted light, and the scale bar is 4 µm.

S9 Figure. *wtf4* coding sequence repeats are dispensable, but can affect antidote rescue.

A. Cartoon of exon 3 and exon 6 coding sequence repeat deletion mutants of *S. kambucha wtf4*. **B-C.** Logo for amino acids encoded by the repeats found in exon 3 (B) and exon 6 (C) of *S. pombe wtf* genes from [20]. The sequence of the amino acids encoded in each repeat region in *S. kambucha wtf4* is shown below each logo. **D.** Spot assay of cells serially diluted and plated on SC-TRP-URA and SC Gal-TRP-URA plates and grown at 30•C for 3 days. Each strain carries both a [*URA3*] and a [*TRP1*] plasmid. The plasmids are either empty (EV) or carry the indicated *wtf4* alleles under the control of galactose-inducible promoters. The horizontal break in the image of each plate is due to rearrangements of the images to facilitate easy comparison. All strains within a panel were grown on the same plates (i.e., one SC-TRP-URA or SC Gal-TRP-URA plate). **E.** Representative images of the same strains as depicted in D were induced in galactose for 4 hours at 30•C to express the indicated Wtf4 proteins. The images are not at the same brightness and contrast settings to clearly show localization of tagged proteins. The arrows in the TL panels highlight vacuoles. 4^{P} indicates Wtf4^{poison}, 4^{A} indicates Wtf4^{antidote}, TL indicates transmitted light, and the scale bar is 4 µm. **F**. Pearson's Correlation between mEos and mCherry signal in cells expressing the specified constructs from E. N>100, ***p<0.001, t-test.

S10 Figure. Coding sequence repeat mutants do not interact with wildtype Wtf4 proteins functionally.

A. Cartoon of coding sequence repeat mutants of *S. kambucha wtf4.* **B-H.** Spot assays of cells serially diluted on SC-TRP-URA and SC Gal-TRP-URA plates and grown at 30•C for 3 days. Each strain carries both a [*URA3*] and a [*TRP1*] plasmid. The plasmids are either empty (EV) or carry the indicated *wtf4* alleles under the control of galactose-inducible promoters. The horizontal breaks in the images in within a panel are due to rearrangements of the images to facilitate easy comparison. All strains within a panel were grown on the same plates (i.e., one SC-TRP-URA or SC Gal-TRP-URA plate in panel B).

bioRxiv preprint doi: https://doi.org/10.1101/2024.08.27.609905; this version posted December 2, 2024. The copyright holder for this preprint (which was not certified by peer review) is the author/funder, who has granted bioRxiv a license to display the preprint in perpetuity. It is made available under aCC-BY 4.0 International license.

S11 Figure. Swapping *wtf* CDS repeats across species affects Wtf25 antidote rescue.

A. Cartoon of a coding sequence repeat mutant of *S. octosporus wtf25*. **B**. Logo representing the amino acids encoded by the repeats found in exon 4 of *S. octosporus wtf* genes from [19]. **C.** The amino acids encoded by the exon 4 repeats of *S. octosporus wtf25* and by the exon 6 repeats of *S. kambucha wtf4*. **D.** Spot assay of cells serially diluted and plated on SC-TRP-URA and SC Gal-TRP-URA plates and grown at 30•C for 3 days. Each strain carries both a [*URA3*] and a [*TRP1*] plasmid. The plasmids are either empty (EV) or carry the indicated *wtf25* alleles under the control of galactose-inducible promoters. The horizontal breaks in the images are due to rearrangements of the images to facilitate easy comparison. All strains within a panel were grown on the same plates (i.e., one SC-TRP-URA or SC Gal-TRP-URA plate). **E.** Representative images of the same strains depicted in D were induced in galactose for 4 hours at 30•C to express the indicated Wtf25 proteins. The images are not at the same brightness and contrast settings to clearly show localization of tagged proteins. The arrows in the TL panel highlight vacuoles. Yellow arrows indicate endoplasmic reticulum-like localization. 25^{P} indicates $Wtf25^{poison}$, 25^{A} indicates $Wtf25^{antidote}$, TL indicates transmitted light, and the scale bar is 4 µm.

S12 Figure. *S. octosporus wtf25* coding sequence repeats are functionally dispensable for poison toxicity but promote antidote rescue.

A. Cartoon of *S. octosporus wtf25* exon 4 coding sequence repeat deletion mutant. **B.** Logo for the amino acids encoded by the repeats found in exon 4 of *S. octosporus wtf* genes from [19] **C.** Spot assay of cells serially diluted and plated on SC-TRP-URA and SC Gal-TRP-URA plates and grown at 30•C for 4 days. Each strain carries both a [*URA3*] and a [*TRP1*] plasmid. The plasmids are either empty (EV) or carry the indicated *wtf25* alleles under the control of galactose-inducible promoters. The horizontal breaks in the images are due to rearrangements of the images to facilitate easy comparison. All strains within a panel were grown on the same plates (i.e., one SC-TRP-URA or SC Gal-TRP-URA plate). **D.** Representative images the same strains depicted in C were induced in galactose for 4 hours at 30•C to express the indicated Wtf25 proteins. The images are not at the same brightness and contrast settings to clearly show localization of tagged proteins. The arrows in the TL panels highlight vacuoles. Yellow arrows indicate endoplasmic reticulum-like localization. 25^{P} indicates $Wtf25^{poison}$, 25^{A} indicates $Wtf25^{antidote}$, TL indicates transmitted light, and the scale bar is 4 µm.

bioRxiv preprint doi: https://doi.org/10.1101/2024.08.27.609905; this version posted December 2, 2024. The copyright holder for this preprint (which was not certified by peer review) is the author/funder, who has granted bioRxiv a license to display the preprint in perpetuity. It is made available under aCC-BY 4.0 International license.

S13 Figure. Idiosyncratic poison and antidote compatibility between wild-type and truncation alleles of *wtf4*.

A. Cartoon of *S. kambucha wtf4* C-terminal truncation mutants. **B.** Spot assay of cells serially diluted and plated on SC-TRP-URA and SC Gal-TRP-URA plates and grown at 30•C for 3 days. Each strain carries both a [*URA3*] and a [*TRP1*] plasmid. The plasmids are either empty (EV) or carry the indicated *wtf4* alleles under the control of galactose-inducible promoters. The horizontal breaks in the images are due to rearrangements of the images to facilitate easy comparison. All strains within a panel were grown on the same plates (i.e., one SC-TRP-URA or SC Gal-TRP-URA plate). **C.** Representative images the strains depicted in B were induced in galactose for 4 hours at 30•C to express the indicated Wtf4 proteins. The images are not at the same brightness and contrast settings to clearly show localization of tagged proteins. The arrows in the TL panels highlight vacuoles. 4^{P} indicates Wtf4^{poison}, 4^{A} indicates Wtf4^{antidote}, TL indicates transmitted light, and the scale bar is 4 µm.

S14 Figure. C-terminus truncation alleles of diverged Wtf^{poison}s are rescued by their corresponding wild type Wtf^{antidote}s.

A. Cartoon of *S. cryophilus wtf1*, *S. osmophilus wtf41*, and *S. octosporus wtf25* C-terminal truncation mutants. **B-D.** Spot assays of cells serially diluted on SC-TRP-URA and SC Gal-TRP-URA plates and grown at 30•C for 3 days. Each strain carries both a [*URA3*] and a [*TRP1*] plasmid. The plasmids are either empty (EV) or carry the indicated *wtf* alleles under the control of galactose-inducible promoters. The horizontal breaks in the images within a panel are due to rearrangements of the images to facilitate easy comparison. All strains within a panel were grown on the same plates (i.e., one SC-TRP-URA or SC Gal-TRP-URA plate).

S1 Table. Overview of *wtf* alleles and their phenotypes in this study. Columns 1-2 describe the allele names and their construction. Columns 3-4 describe the toxicity of the poison and antidote proteins encoded by the corresponding allele. Column 5 notes if the mutant antidote can rescue the corresponding poison allele. Column 6-7 note if the specific antidote allele can rescue the wild-type poison (Column 6), or if the specific poison can be rescued by the wild-type antidote (Column 7). Column 9 notes if the corresponding antidote can rescue other poison alleles. Column 10 notes the AmFRET values are comparable to the wild-type Wtf4^{poison}-mEos or monomer-mEos. Finally, column 11 notes the figures/supplemental figures where these

bioRxiv preprint doi: https://doi.org/10.1101/2024.08.27.609905; this version posted December 2, 2024. The copyright holder for this preprint (which was not certified by peer review) is the author/funder, who has granted bioRxiv a license to display the preprint in perpetuity. It is made available under aCC-BY 4.0 International license.

alleles can be found. Rows are separated based on the wild-type allele that the mutants were constructed from, where applicable. ND indicates that the specified experiment was not done.

S2 Table. Yeast strains used in this study. Column 1 is the figure where these strains were used, Column 2 is the name of the yeast strain and column 3 is the genotype of the strain. If the strain was constructed in this study, column 4 has the details on how the strain was constructed. If the strain was constructed in another study, column 5 notes the references for the same.

S3 Table. Plasmids used in this study. Column 1 denotes if the plasmids were used for strain construction or cloning, and the specific wild-type alleles that the plasmids correspond to. Column 2 is the name of the plasmid and column 3 is a short description of the plasmid. If the plasmid was not constructed in this study, column 4 references the studies the plasmids were constructed in.

S4 Table. Oligos used in this study. Column 1 denotes the name of the oligo and column 2 has the sequence of the oligos. If the oligo was not constructed in this study, column 3 references the studies the oligos were used in.

S5 Table. Overview of features across *wtf* driver genes. Column 1 denotes the *wtf* driver gene, and columns 2-8 describe specific features of each gene and their encoded proteins. Column 2 notes if the gene has been shown to drive in fission yeast or been shown to encode for functional Wtf poison and antidote proteins in *S. cerevisiae*. Column 3 notes the number of exons in the gene, and column 4 notes the size of the $Wtf^{antidote}$ protein in amino acids. If the antidote sequence had a predicted coiled-coil domain, column 4 notes the size and location in the antidote protein as described in [15]. Column 5 notes the numbers of PY motifs (L/PPXY; [24]) in exon 1. While some $Wtf^{antidote}$ proteins also have an additional PY motif in exon 2, we didn't include that information here since those motifs are shared by the corresponding poison proteins as well. Column 6 notes the number of predicted transmembrane domains predicted by TMHMM2.0 [71,72]. Column 7 notes the length of the exon 3 coding sequence repeats (if present) in amino acids. Column 8 notes the length of the exon 4/6 coding sequence repeats (if present) in amino acids. NA (not applicable) here indicates that the repeats were not present in the Wtf protein. Column 8 mentions the reference(s) where these genes/encoded proteins were characterized.

bioRxiv preprint doi: https://doi.org/10.1101/2024.08.27.609905; this version posted December 2, 2024. The copyright holder for this preprint (which was not certified by peer review) is the author/funder, who has granted bioRxiv a license to display the preprint in perpetuity. It is made available under aCC-BY 4.0 International license.

S6 Table. Overview of predicted transmembrane domains of Wtf^{antidote} proteins. The results from the TMHMM2.0 [71,72] analysis of Wtf^{antidote} proteins encoded by *wtf* driver genes in S5 table are detailed here. Find the specific results of the analyses, including the number of amino acids in the predicted transmembrane domains, probability of the N-terminal being internal to the membrane (N-in), the specific location and the length of the predicted transmembrane domains.

S1 Data. Raw Data for Pearson's Correlation tests performed in this study. Each sheet contains the raw data for the Pearson's Correlation tests and the statistical analysis corresponding to the tests performed in Fig 4F, S4 Fig E, Fig 6F and S9 Fig F respectively. See Methods for how this analysis was performed.

S2 Data. Raw Data for AmFRET plots presented in this study. Each sheet contains the raw data and the statistical analysis for the AmFRET plots of different Wtf^{poison}-mEos proteins, Wtf4^{antidote}-mEos, *S. kambucha wtf4* Exon1-mEos and monomer-mEos proteins in Fig 2B, S2 Fig E, S3 Fig D, Fig 4C, S5 Fig, S6 Fig D and S7 Fig B respectively. The corresponding raw data for the Acceptor fluorescence intensity presented in S6 Fig for each of these experiments are found next to the relevant AmFRET data. See Methods for how this analysis was performed.

S1 Figure

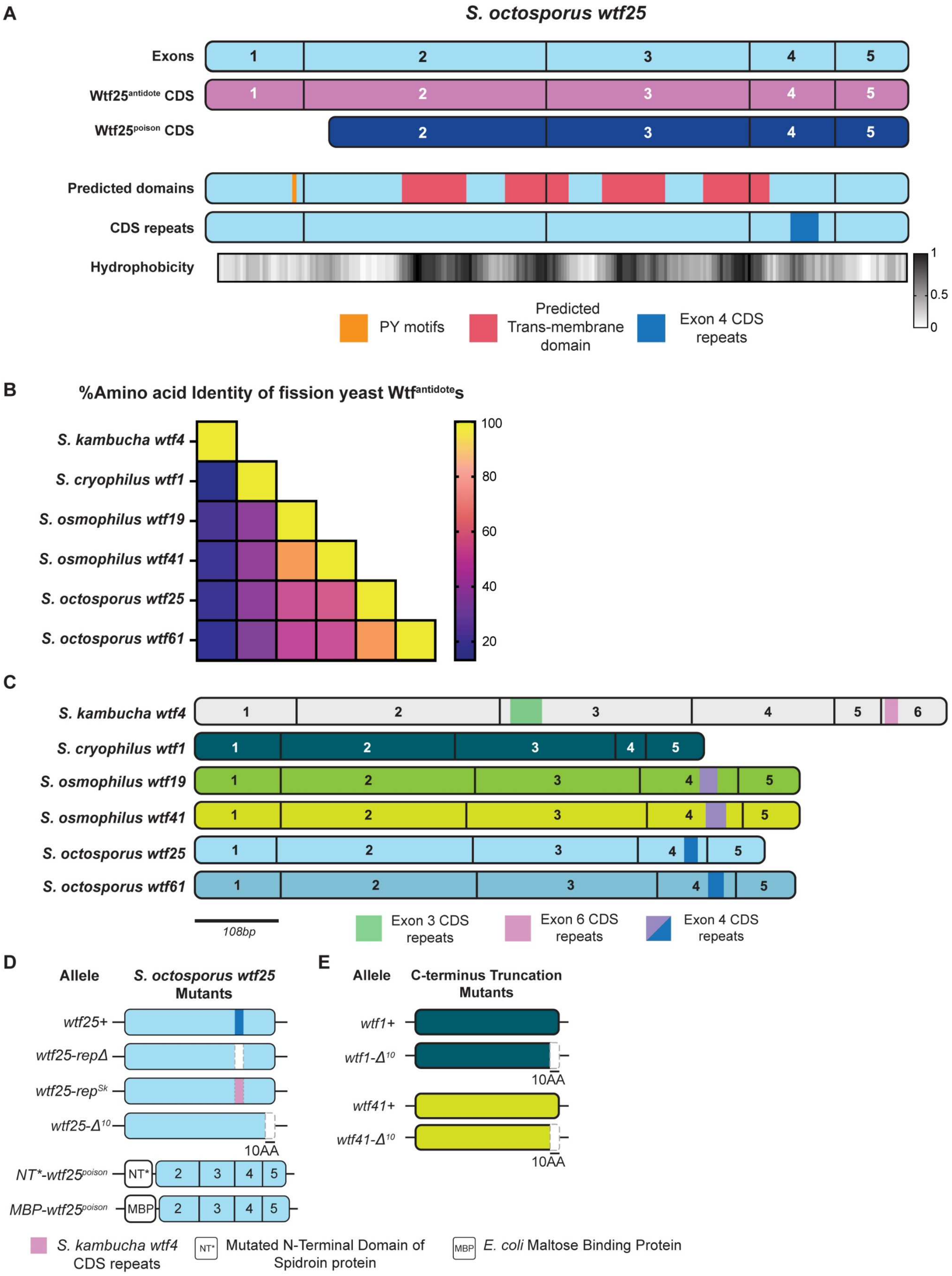
A
S. octosporus wtf25
Exons
Wtf25antidote CDS
Wtf25poison CDS
1
2
3
4
5
Predicted domains
CDS repeats
Hydrophobicity
1
0.5
0
PY motifs
Predicted Trans-membrane domain
Exon 4 CDS repeats
B
%Amino acid Identity of fission yeast Wtfantidotes
S. kambucha wtf4
S. cryophilus wtf1
S. osmophilus wtf19
S. osmophilus wtf41
S. octosporus wtf25
S. octosporus wtf61
100
80
60
40
20
C
S. kambucha wtf4
S. cryophilus wtf1
S. osmophilus wtf19
S. osmophilus wtf41
S. octosporus wtf25
S. octosporus wtf61
6
108bp
Exon 3 CDS repeats
Exon 6 CDS repeats
Exon 4 CDS repeats
D
Allele
S. octosporus wtf25 Mutants
wtf25+
wtf25-repΔ
wtf25-repSk
wtf25-Δ10
10AA
NT*-wtf25poison
MBP-wtf25poison
NT*
MBP
E
Allele
C-terminus Truncation Mutants
wtf1+
wtf1-Δ10
10AA
wtf41+
wtf41-Δ10
10AA
S. kambucha wtf4 CDS repeats
NT* Mutated N-Terminal Domain of Spidroin protein
MBP E. coli Maltose Binding Protein

S2 Figure

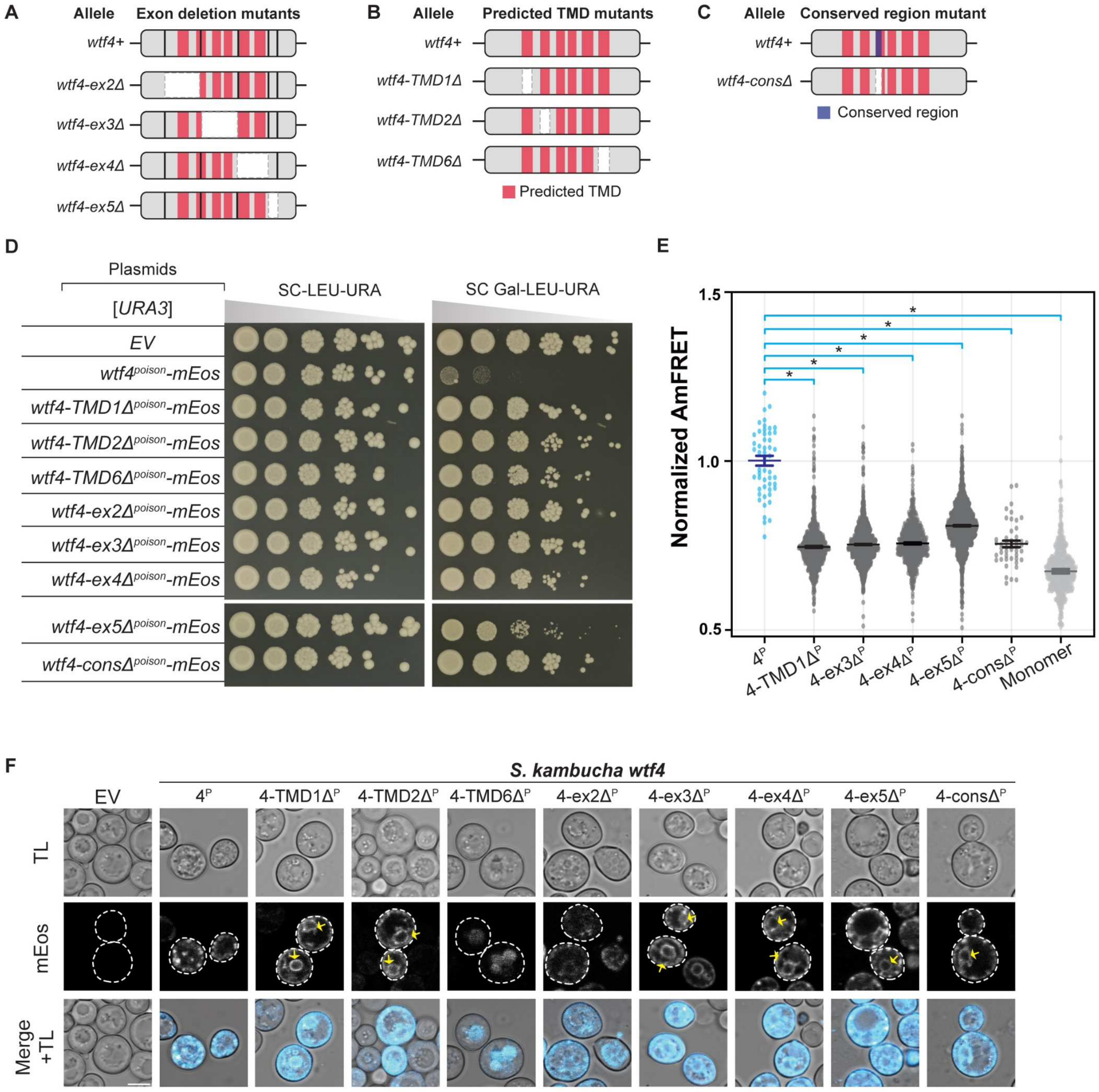
A
Allele
Exon deletion mutants
wtf4+
wtf4-ex2Δ
wtf4-ex3Δ
wtf4-ex4Δ
wtf4-ex5Δ
B
Allele
Predicted TMD mutants
wtf4+
wtf4-TMD1Δ
wtf4-TMD2Δ
wtf4-TMD6Δ
Predicted TMD
C
Allele
Conserved region mutant
wtf4+
wtf4-consΔ
Conserved region
D
Plasmids
[URA3]
SC-LEU-URA
SC Gal-LEU-URA
EV
wtf4poison-mEos
wtf4-TMD1Δpoison-mEos
wtf4-TMD2Δpoison-mEos
wtf4-TMD6Δpoison-mEos
wtf4-ex2Δpoison-mEos
wtf4-ex3Δpoison-mEos
wtf4-ex4Δpoison-mEos
wtf4-ex5Δpoison-mEos
wtf4-consΔpoison-mEos
E
Normalized AmFRET
1.5
1.0
0.5
*
4P
4-TMD1ΔP
4-ex3ΔP
4-ex4ΔP
4-ex5ΔP
4-consΔP
Monomer
F
S. kambucha wtf4
EV
4P
4-TMD1ΔP
4-TMD2ΔP
4-TMD6ΔP
4-ex2ΔP
4-ex3ΔP
4-ex4ΔP
4-ex5ΔP
4-consΔP
TL
mEos
Merge +TL

S3 Figure

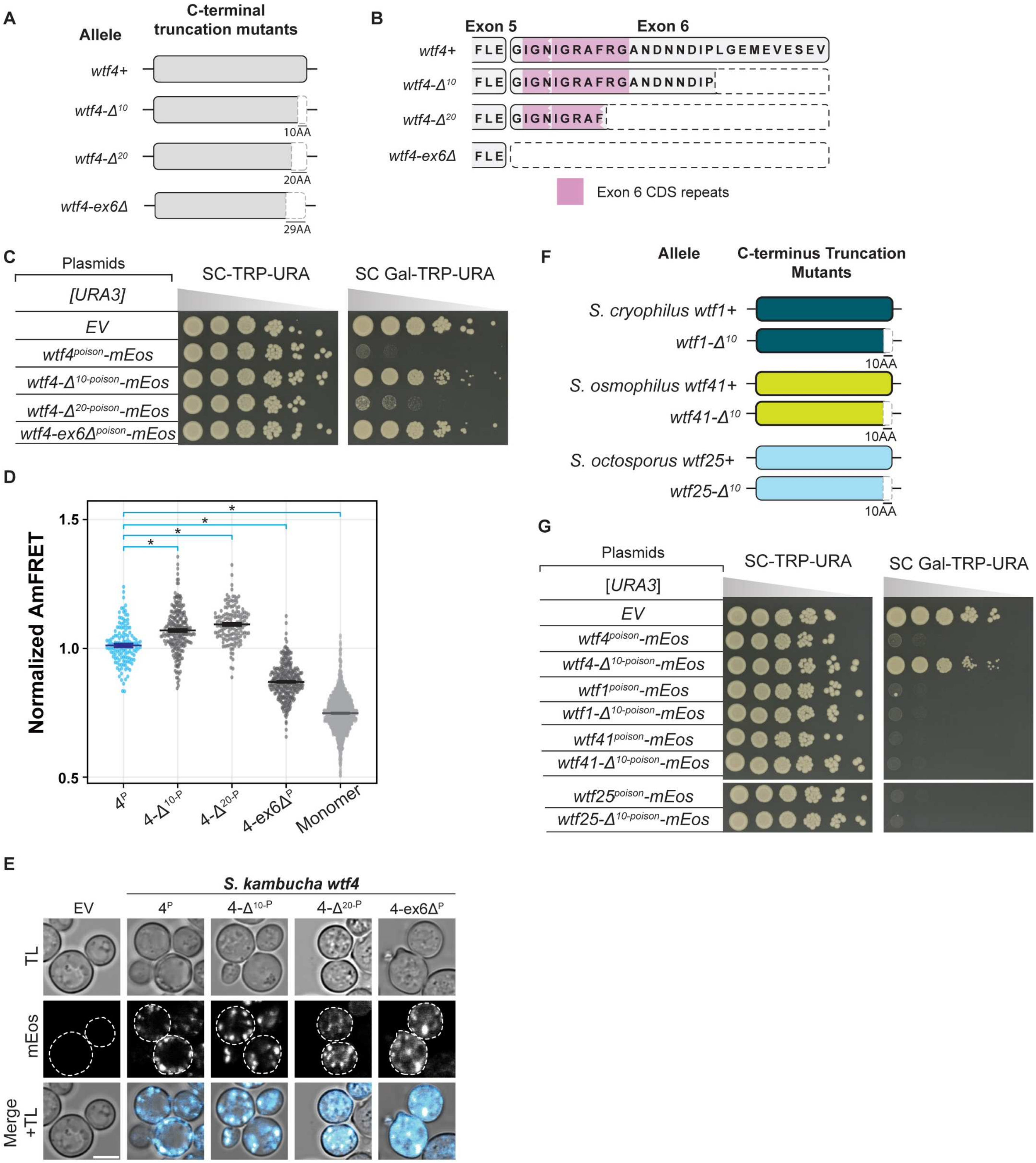
A
Allele
C-terminal truncation mutants
wtf4+
wtf4-Δ10
wtf4-Δ20
wtf4-ex6Δ
10AA
20AA
29AA
B
Exon 5
Exon 6
FLE
GIGNIGRAFRGANDNNDIPLGEMEVESEV
GIGNIGRAFRGANDNNDIP
GIGNIGRAF
Exon 6 CDS repeats
C
Plasmids
[URA3]
SC-TRP-URA
SC Gal-TRP-URA
EV
wtf4poison-mEos
wtf4-Δ10-poison-mEos
wtf4-Δ20-poison-mEos
wtf4-ex6Δpoison-mEos
D
Normalized AmFRET
1.5
1.0
0.5
4P
4-Δ10-P
4-Δ20-P
4-ex6ΔP
Monomer
E
S. kambucha wtf4
EV
TL
mEos
Merge +TL
F
Allele
C-terminus Truncation Mutants
S. cryophilus wtf1+
wtf1-Δ10
S. osmophilus wtf41+
wtf41-Δ10
S. octosporus wtf25+
wtf25-Δ10
G
wtf1poison-mEos
wtf1-Δ10-poison-mEos
wtf41poison-mEos
wtf41-Δ10-poison-mEos
wtf25poison-mEos
wtf25-Δ10-poison-mEos

S4 Figure

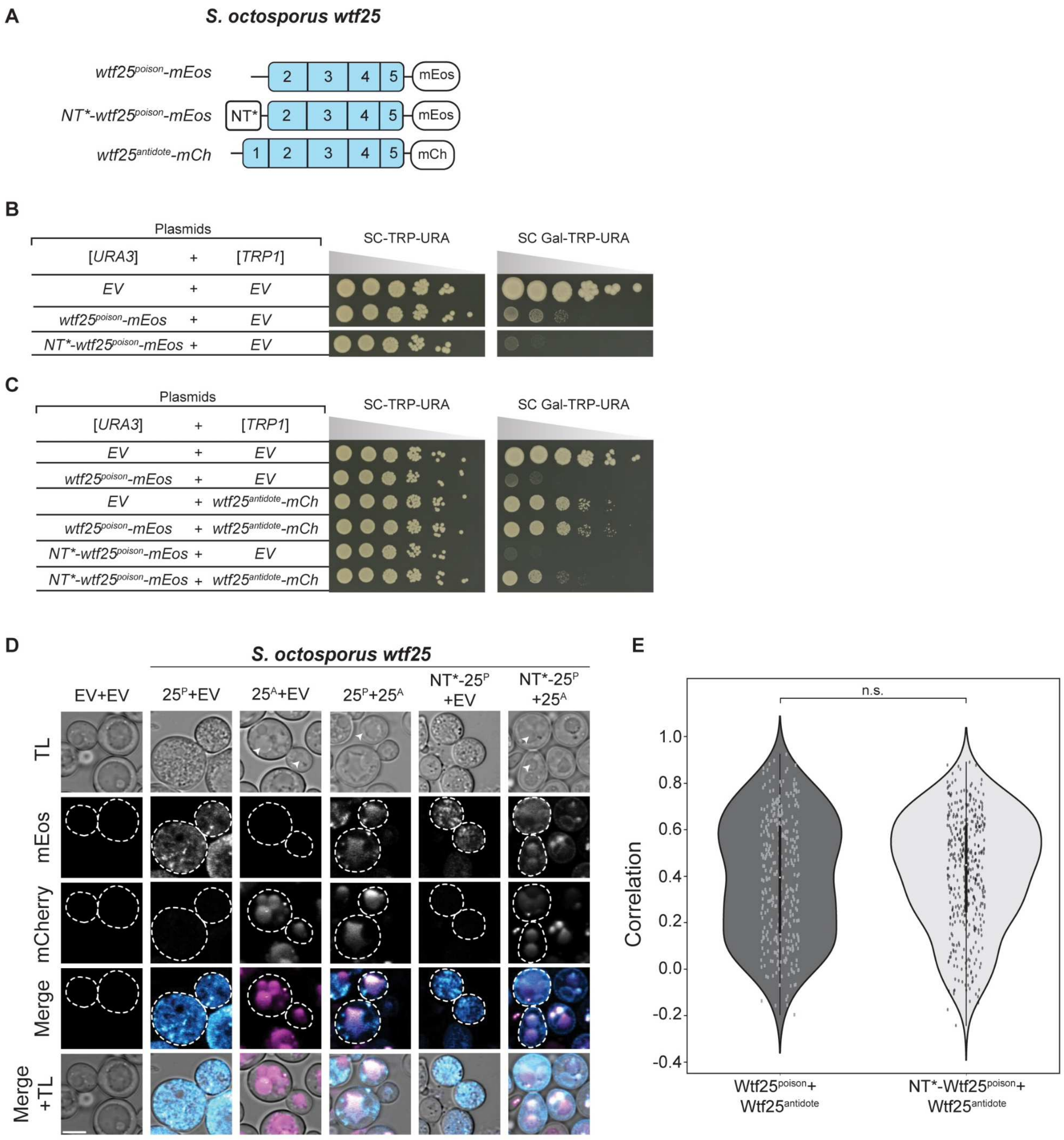

A
S. octosporus wtf25
$wtf25^{poison}$-mEos
NT^{*}-$wtf25^{poison}$-mEos
$wtf25^{antidote}$-mCh
NT*
1
2
3
4
5
mEos
mCh
B
Plasmids
[URA3]
+
[TRP1]
SC-TRP-URA
SC Gal-TRP-URA
EV + EV
$wtf25^{poison}$-mEos + EV
NT^{*}-$wtf25^{poison}$-mEos + EV
C
Plasmids
[URA3]
+
[TRP1]
SC-TRP-URA
SC Gal-TRP-URA
EV + EV
$wtf25^{poison}$-mEos + EV
EV + $wtf25^{antidote}$-mCh
$wtf25^{poison}$-mEos + $wtf25^{antidote}$-mCh
NT^{*}-$wtf25^{poison}$-mEos + EV
NT^{*}-$wtf25^{poison}$-mEos + $wtf25^{antidote}$-mCh
D
S. octosporus wtf25
EV+EV
25^{P}+EV
25^{A}+EV
25^{P}+25^{A}
NT*-25^{P} +EV
NT*-25^{P} +25^{A}
TL
mEos
mCherry
Merge
Merge +TL
E
n.s.
Correlation
1.0
0.8
0.6
0.4
0.2
0.0
-0.2
-0.4
$Wtf25^{poison}$+ $Wtf25^{antidote}$
NT*-$Wtf25^{poison}$+ $Wtf25^{antidote}$

S5 Figure

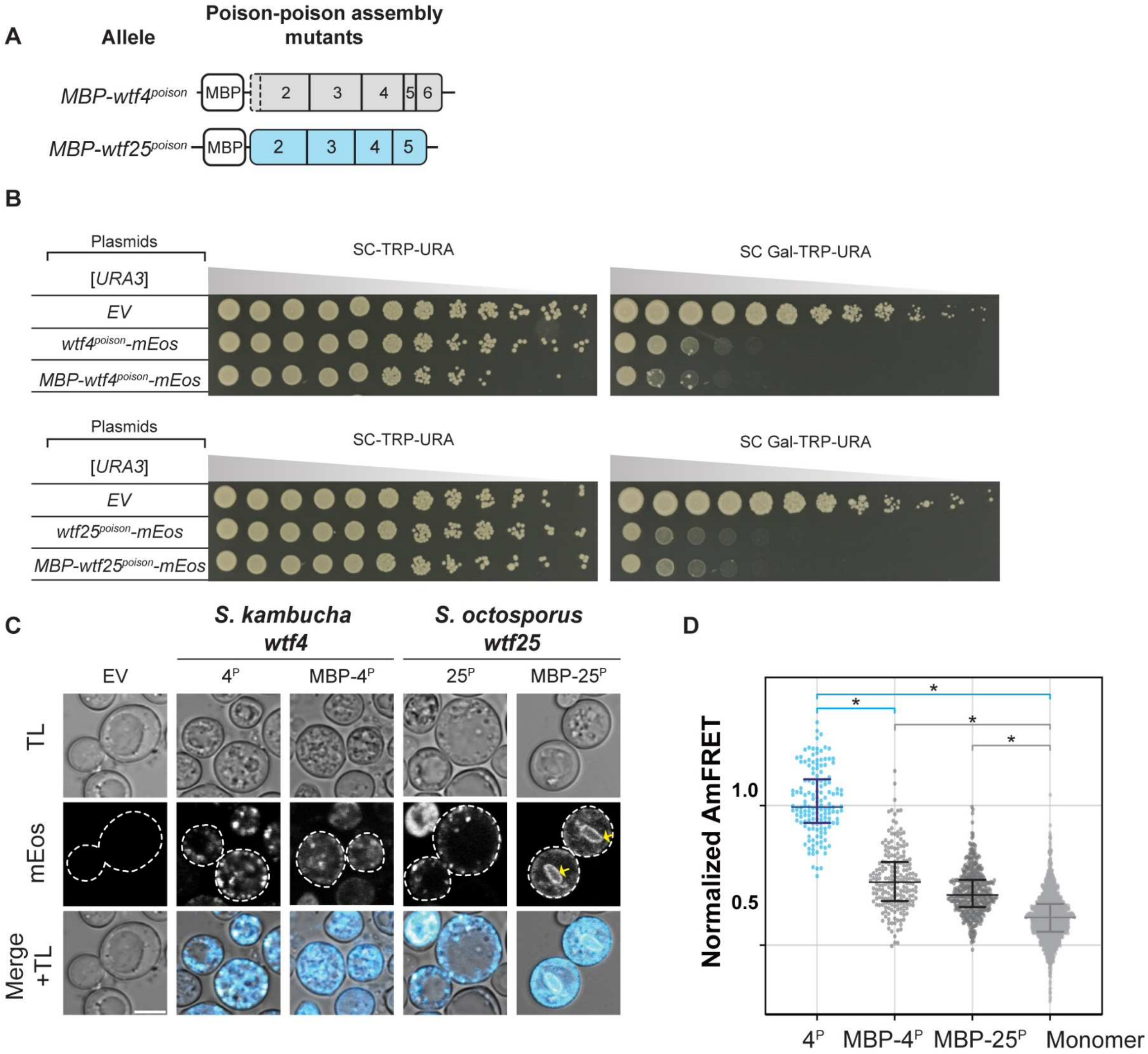
A
Allele
Poison-poison assembly mutants
MBP-wtf4poison
MBP
2
3
4
5
6
MBP-wtf25poison
MBP
2
3
4
5
B
Plasmids
[URA3]
EV
wtf4poison-mEos
MBP-wtf4poison-mEos
SC-TRP-URA
SC Gal-TRP-URA
Plasmids
[URA3]
EV
wtf25poison-mEos
MBP-wtf25poison-mEos
SC-TRP-URA
SC Gal-TRP-URA
C
S. kambucha wtf4
S. octosporus wtf25
EV
4P
MBP-4P
25P
MBP-25P
TL
mEos
Merge +TL
D
Normalized AmFRET
1.0
0.5
*
*
*
*
4P
MBP-4P
MBP-25P
Monomer

S6 Figure

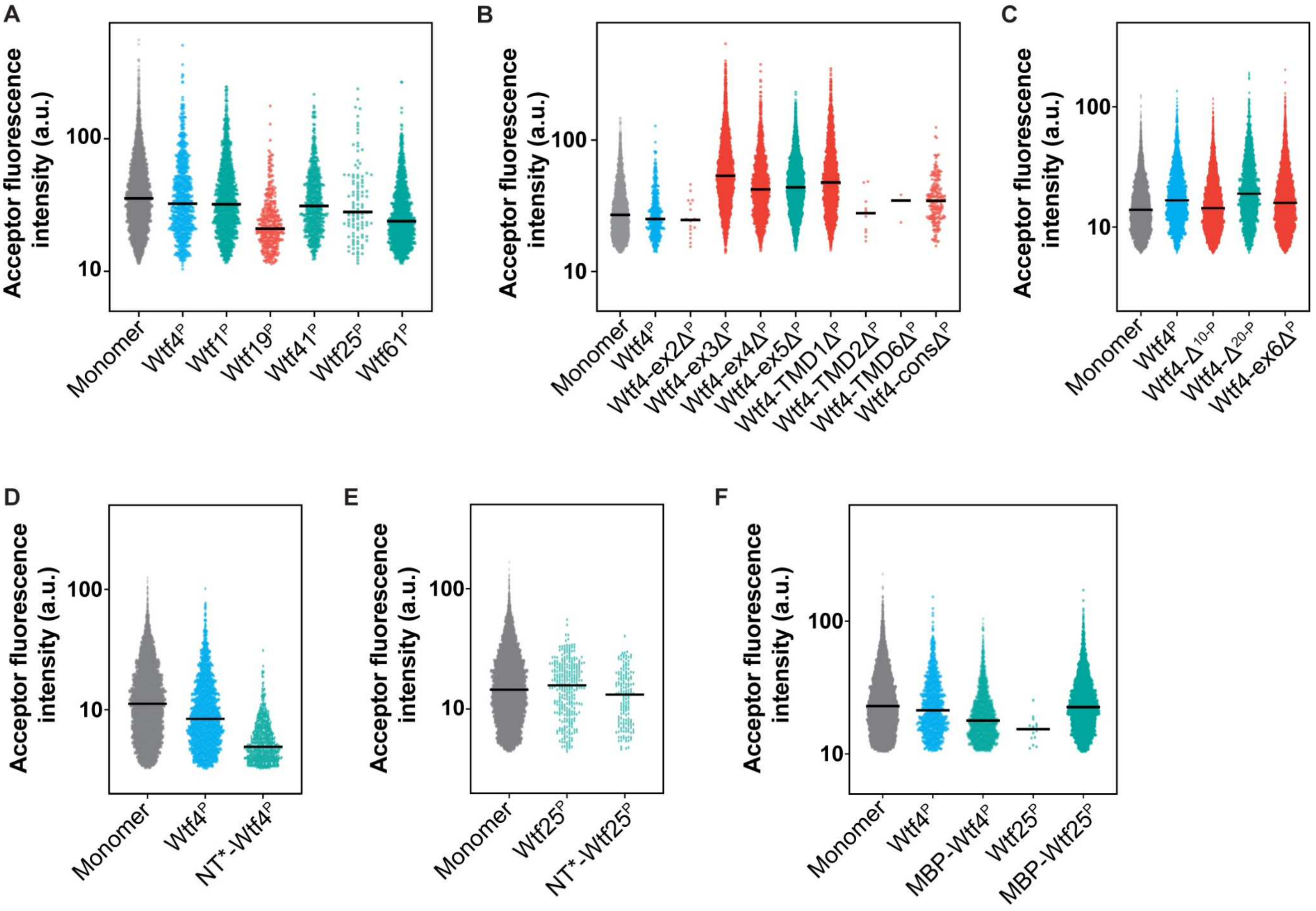

A
Acceptor fluorescence intensity (a.u.)
100
10
Monomer
Wtf4P
Wtf1P
Wtf19P
Wtf41P
Wtf25P
Wtf61P
B
Acceptor fluorescence intensity (a.u.)
100
10
Monomer
Wtf4P
Wtf4-ex2ΔP
Wtf4-ex3ΔP
Wtf4-ex4ΔP
Wtf4-ex5ΔP
Wtf4-TMD1ΔP
Wtf4-TMD2ΔP
Wtf4-TMD6ΔP
Wtf4-consΔP
C
Acceptor fluorescence intensity (a.u.)
100
10
Monomer
Wtf4P
Wtf4-Δ10-P
Wtf4-Δ20-P
Wtf4-ex6ΔP
D
Acceptor fluorescence intensity (a.u.)
100
10
Monomer
Wtf4P
NT*-Wtf4P
E
Acceptor fluorescence intensity (a.u.)
100
10
Monomer
Wtf25P
NT*-Wtf25P
F
Acceptor fluorescence intensity (a.u.)
100
10
Monomer
Wtf4P
MBP-Wtf4P
Wtf25P
MBP-Wtf25P

S7 Figure

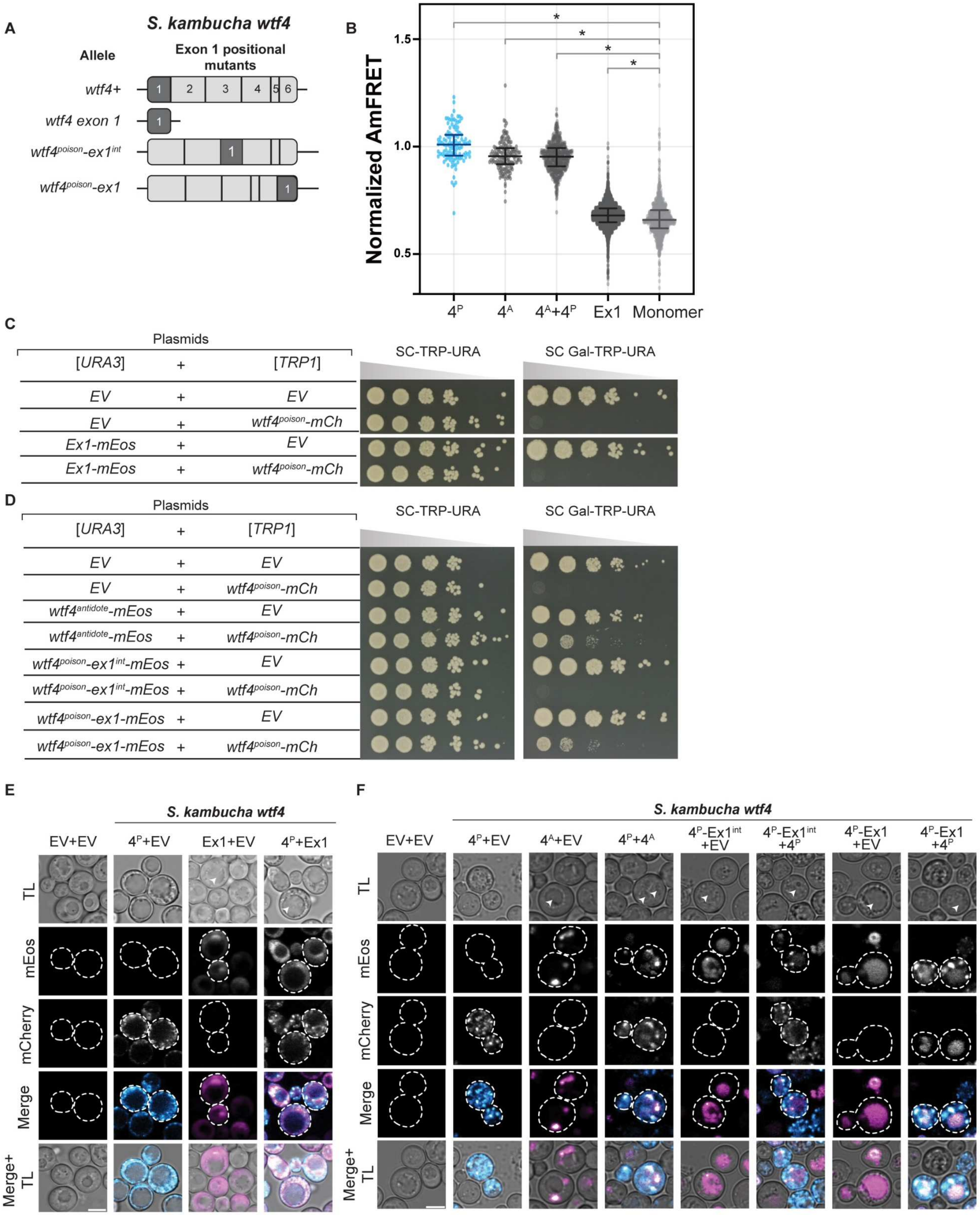

A
S. kambucha wtf4
Allele
Exon 1 positional mutants
wtf4+
wtf4 exon 1
wtf4poison-ex1int
wtf4poison-ex1
B
Normalized AmFRET
4P
4A
4A+4P
Ex1
Monomer
C
Plasmids
[URA3] + [TRP1]
SC-TRP-URA
SC Gal-TRP-URA
EV + EV
EV + wtf4poison-mCh
Ex1-mEos + EV
Ex1-mEos + wtf4poison-mCh
D
Plasmids
[URA3] + [TRP1]
SC-TRP-URA
SC Gal-TRP-URA
EV + EV
EV + wtf4poison-mCh
wtf4antidote-mEos + EV
wtf4antidote-mEos + wtf4poison-mCh
wtf4poison-ex1int-mEos + EV
wtf4poison-ex1int-mEos + wtf4poison-mCh
wtf4poison-ex1-mEos + EV
wtf4poison-ex1-mEos + wtf4poison-mCh
E
S. kambucha wtf4
EV+EV
4P+EV
Ex1+EV
4P+Ex1
TL
mEos
mCherry
Merge
Merge+ TL
F
S. kambucha wtf4
EV+EV
4P+EV
4A+EV
4P+4A
4P-Ex1int +EV
4P-Ex1int +4P
4P-Ex1 +EV
4P-Ex1 +4P
TL
mEos
mCherry
Merge
Merge+ TL

S8 Figure

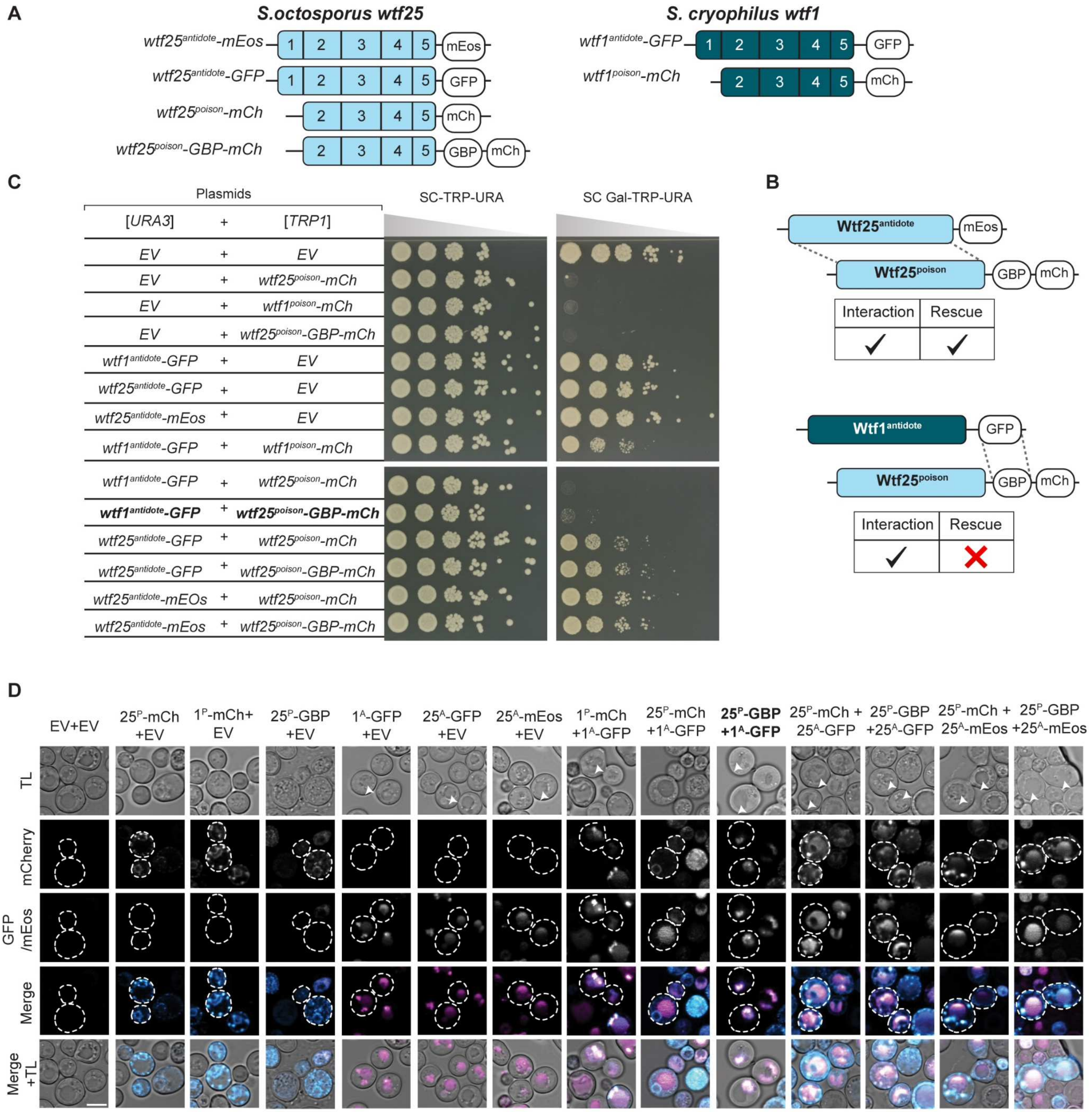
A
S.octosporus wtf25
wtf25antidote-mEos
wtf25antidote-GFP
wtf25poison-mCh
wtf25poison-GBP-mCh
S. cryophilus wtf1
wtf1antidote-GFP
wtf1poison-mCh
B
Wtf25antidote
Wtf25poison
Wtf1antidote
Interaction
Rescue
C
Plasmids
[URA3] + [TRP1]
SC-TRP-URA
SC Gal-TRP-URA
EV + EV
EV + wtf25poison-mCh
EV + wtf1poison-mCh
EV + wtf25poison-GBP-mCh
wtf1antidote-GFP + EV
wtf25antidote-GFP + EV
wtf25antidote-mEos + EV
wtf1antidote-GFP + wtf1poison-mCh
wtf1antidote-GFP + wtf25poison-mCh
wtf1antidote-GFP + wtf25poison-GBP-mCh
wtf25antidote-GFP + wtf25poison-mCh
wtf25antidote-GFP + wtf25poison-GBP-mCh
wtf25antidote-mEOs + wtf25poison-mCh
wtf25antidote-mEos + wtf25poison-GBP-mCh
D
EV+EV
25P-mCh +EV
1P-mCh+ EV
25P-GBP +EV
1A-GFP +EV
25A-GFP +EV
25A-mEos +EV
1P-mCh +1A-GFP
25P-mCh +1A-GFP
25P-GBP +1A-GFP
25P-mCh + 25A-GFP
25P-GBP +25A-GFP
25P-mCh + 25A-mEos
25P-GBP +25A-mEos
TL
mCherry
GFP /mEos
Merge
Merge +TL

S9 Figure

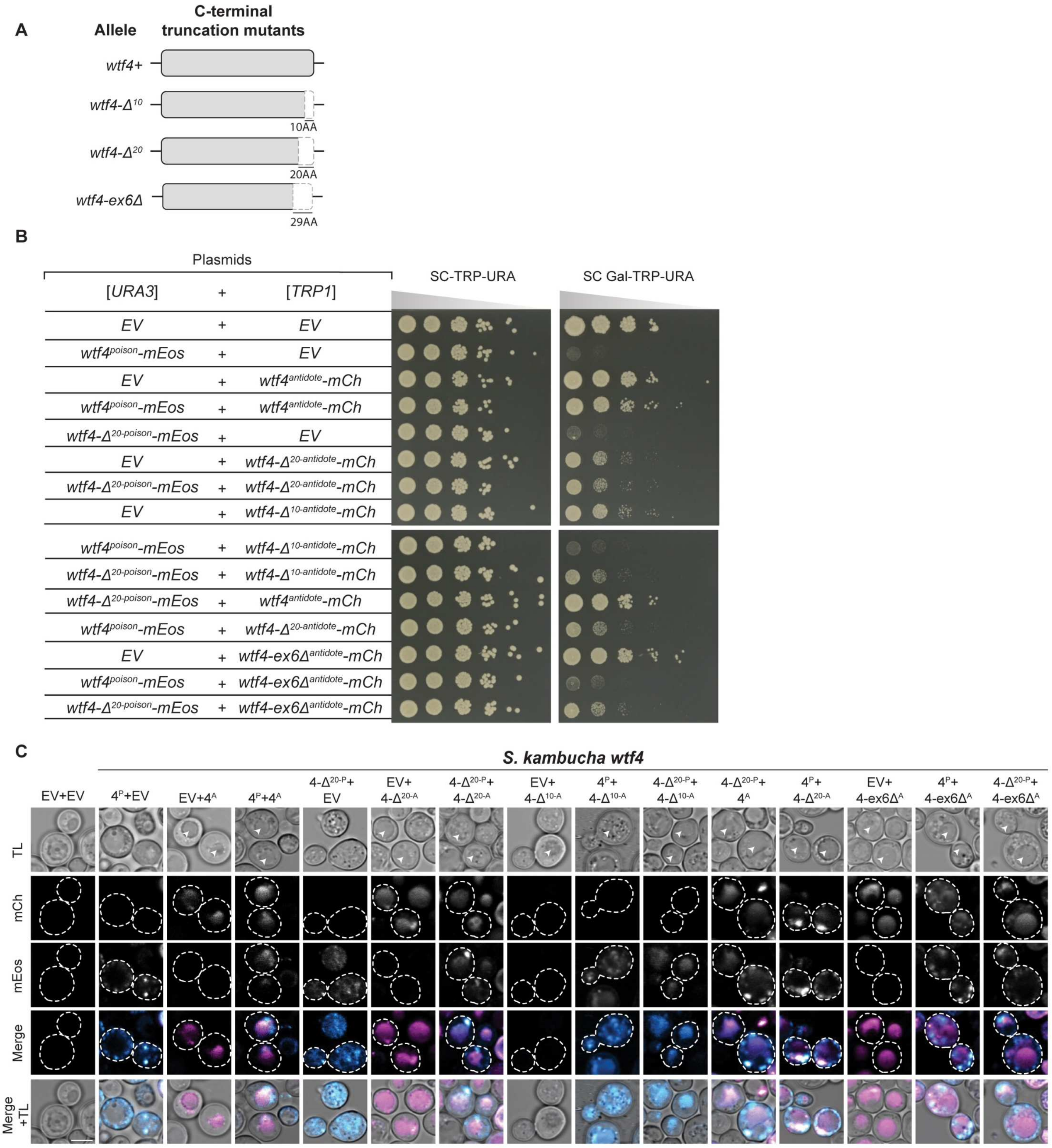
A
Allele
C-terminal truncation mutants
wtf4+
wtf4-Δ^{10}
10AA
wtf4-Δ^{20}
20AA
wtf4-ex6Δ
29AA
B
Plasmids
[URA3]
+
[TRP1]
SC-TRP-URA
SC Gal-TRP-URA
EV + EV
$wtf4^{poison}$-mEos + EV
EV + $wtf4^{antidote}$-mCh
$wtf4^{poison}$-mEos + $wtf4^{antidote}$-mCh
wtf4-$\Delta^{20-poison}$-mEos + EV
EV + wtf4-$\Delta^{20-antidote}$-mCh
wtf4-$\Delta^{20-poison}$-mEos + wtf4-$\Delta^{20-antidote}$-mCh
EV + wtf4-$\Delta^{10-antidote}$-mCh
$wtf4^{poison}$-mEos + wtf4-$\Delta^{10-antidote}$-mCh
wtf4-$\Delta^{20-poison}$-mEos + wtf4-$\Delta^{10-antidote}$-mCh
wtf4-$\Delta^{20-poison}$-mEos + $wtf4^{antidote}$-mCh
$wtf4^{poison}$-mEos + wtf4-$\Delta^{20-antidote}$-mCh
EV + wtf4-ex6$\Delta^{antidote}$-mCh
$wtf4^{poison}$-mEos + wtf4-ex6$\Delta^{antidote}$-mCh
wtf4-$\Delta^{20-poison}$-mEos + wtf4-ex6$\Delta^{antidote}$-mCh
C
S. kambucha wtf4
EV+EV
4^{P}+EV
EV+4^{A}
4^{P}+4^{A}
4-Δ^{20-P}+ EV
EV+ 4-Δ^{20-A}
4-Δ^{20-P}+ 4-Δ^{20-A}
EV+ 4-Δ^{10-A}
4^{P}+ 4-Δ^{10-A}
4-Δ^{20-P}+ 4-Δ^{10-A}
4-Δ^{20-P}+ 4^{A}
4^{P}+ 4-Δ^{20-A}
EV+ 4-ex6Δ^{A}
4^{P}+ 4-ex6Δ^{A}
4-Δ^{20-P}+ 4-ex6Δ^{A}
TL
mCh
mEos
Merge
Merge +TL

S10 Figure

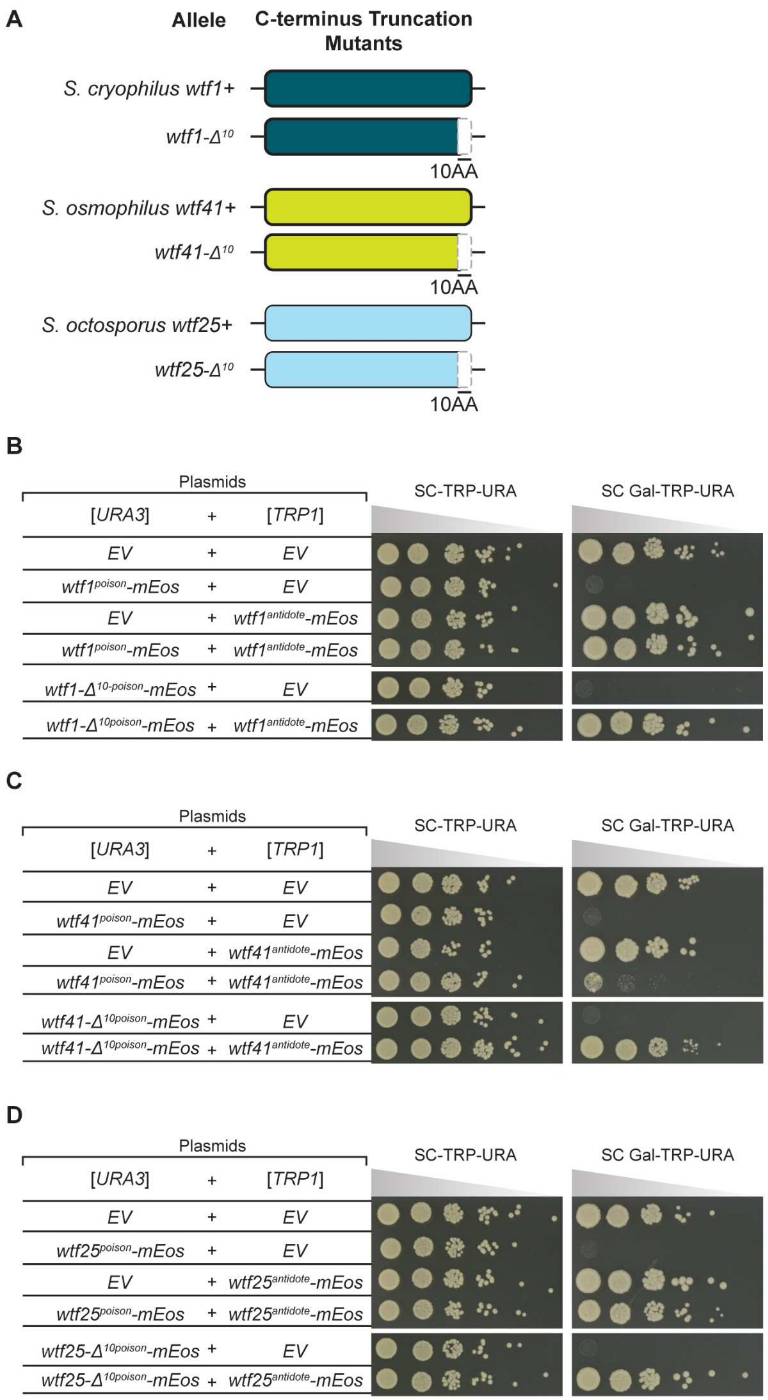
A
Allele
C-terminus Truncation Mutants
S. cryophilus wtf1+
wtf1-Δ10
10AA
S. osmophilus wtf41+
wtf41-Δ10
10AA
S. octosporus wtf25+
wtf25-Δ10
10AA
B
Plasmids
[URA3] + [TRP1]
SC-TRP-URA
SC Gal-TRP-URA
EV + EV
wtf1poison-mEos + EV
EV + wtf1antidote-mEos
wtf1poison-mEos + wtf1antidote-mEos
wtf1-Δ10-poison-mEos + EV
wtf1-Δ10poison-mEos + wtf1antidote-mEos
C
Plasmids
[URA3] + [TRP1]
SC-TRP-URA
SC Gal-TRP-URA
EV + EV
wtf41poison-mEos + EV
EV + wtf41antidote-mEos
wtf41poison-mEos + wtf41antidote-mEos
wtf41-Δ10poison-mEos + EV
wtf41-Δ10poison-mEos + wtf41antidote-mEos
D
Plasmids
[URA3] + [TRP1]
SC-TRP-URA
SC Gal-TRP-URA
EV + EV
wtf25poison-mEos + EV
EV + wtf25antidote-mEos
wtf25poison-mEos + wtf25antidote-mEos
wtf25-Δ10poison-mEos + EV
wtf25-Δ10poison-mEos + wtf25antidote-mEos

S11 Figure

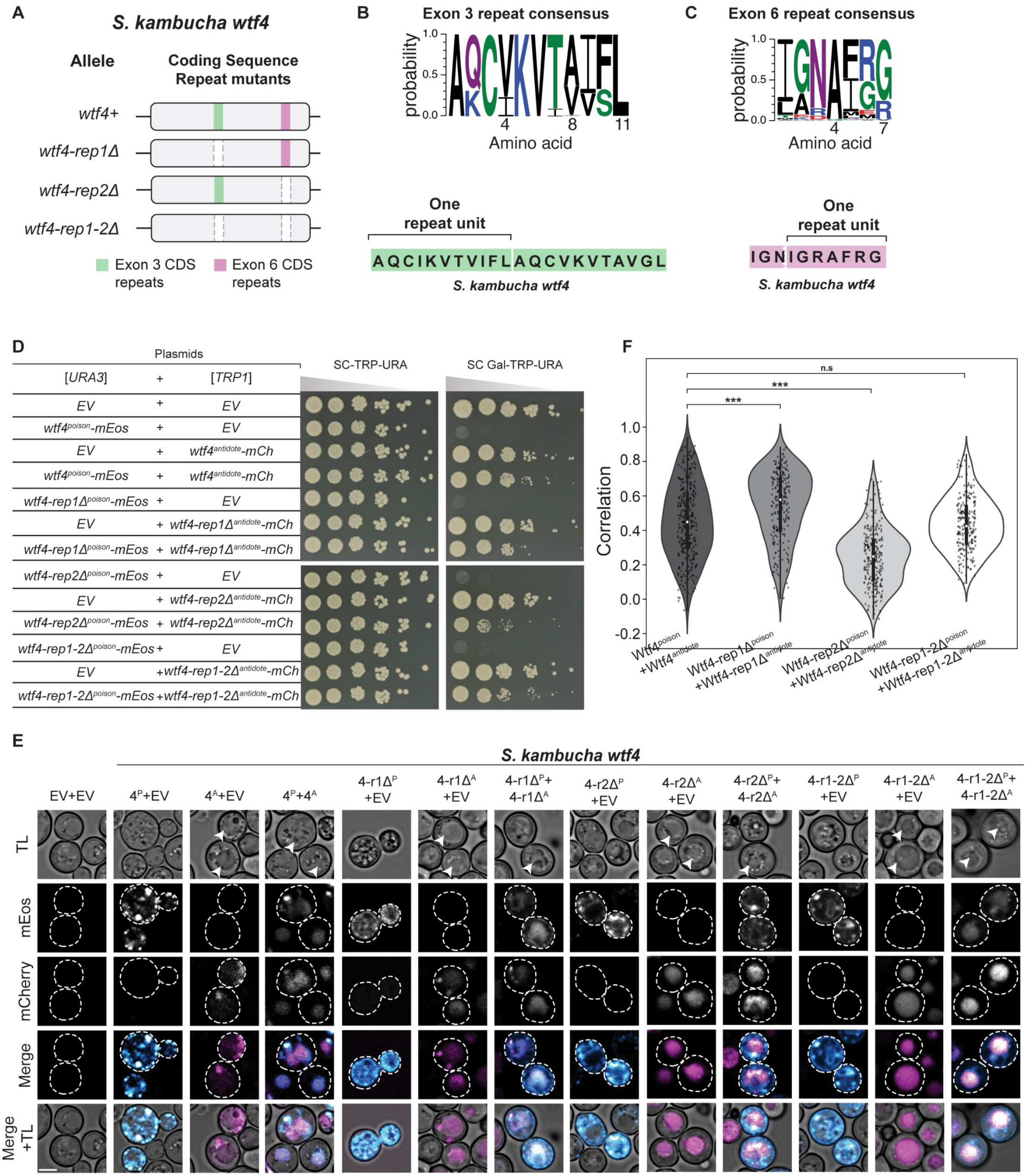
A
S. kambucha wtf4
Allele
Coding Sequence Repeat mutants
wtf4+
wtf4-rep1Δ
wtf4-rep2Δ
wtf4-rep1-2Δ
Exon 3 CDS repeats
Exon 6 CDS repeats
B
Exon 3 repeat consensus
probability
Amino acid
One repeat unit
AQCIKVTVIFL AQCVKVTAVGL
S. kambucha wtf4
C
Exon 6 repeat consensus
One repeat unit
IGN IGRAFRG
D
Plasmids
[URA3] + [TRP1]
SC-TRP-URA
SC Gal-TRP-URA
E
S. kambucha wtf4
TL
mEos
mCherry
Merge
Merge +TL
F
Correlation
n.s
***

S12 Figure

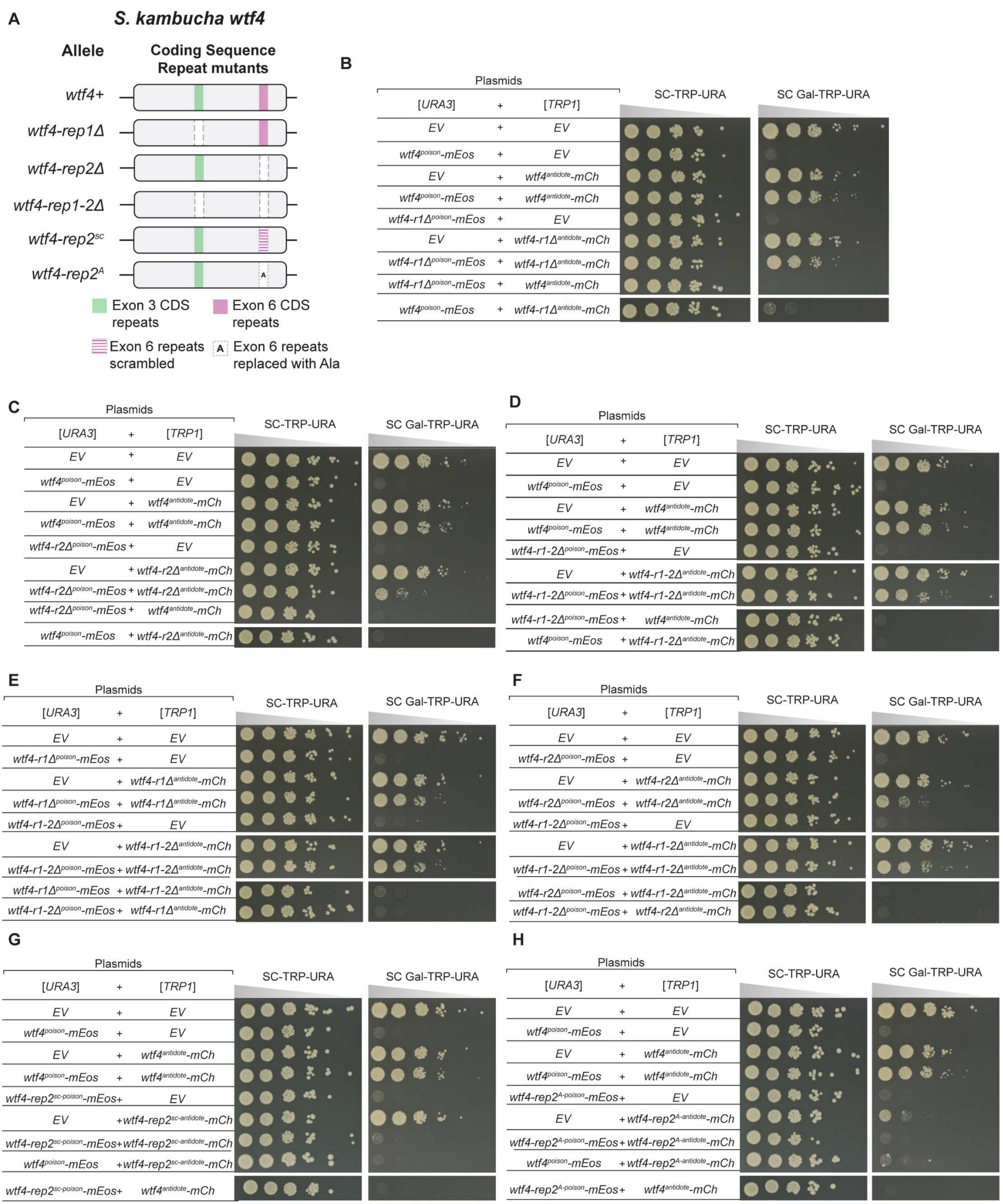
A
S. kambucha wtf4
Allele
Coding Sequence Repeat mutants
wtf4+
wtf4-rep1Δ
wtf4-rep2Δ
wtf4-rep1-2Δ
wtf4-rep2sc
wtf4-rep2A
Exon 3 CDS repeats
Exon 6 CDS repeats
Exon 6 repeats scrambled
Exon 6 repeats replaced with Ala
B
C
D
E
F
G
H
Plasmids
[URA3] + [TRP1]
SC-TRP-URA
SC Gal-TRP-URA
EV
wtf4poison-mEos
wtf4antidote-mCh
wtf4-r1Δpoison-mEos
wtf4-r1Δantidote-mCh
wtf4-r2Δpoison-mEos
wtf4-r2Δantidote-mCh
wtf4-r1-2Δpoison-mEos
wtf4-r1-2Δantidote-mCh
wtf4-rep2sc-poison-mEos
wtf4-rep2sc-antidote-mCh
wtf4-rep2A-poison-mEos
wtf4-rep2A-antidote-mCh

S13 Figure

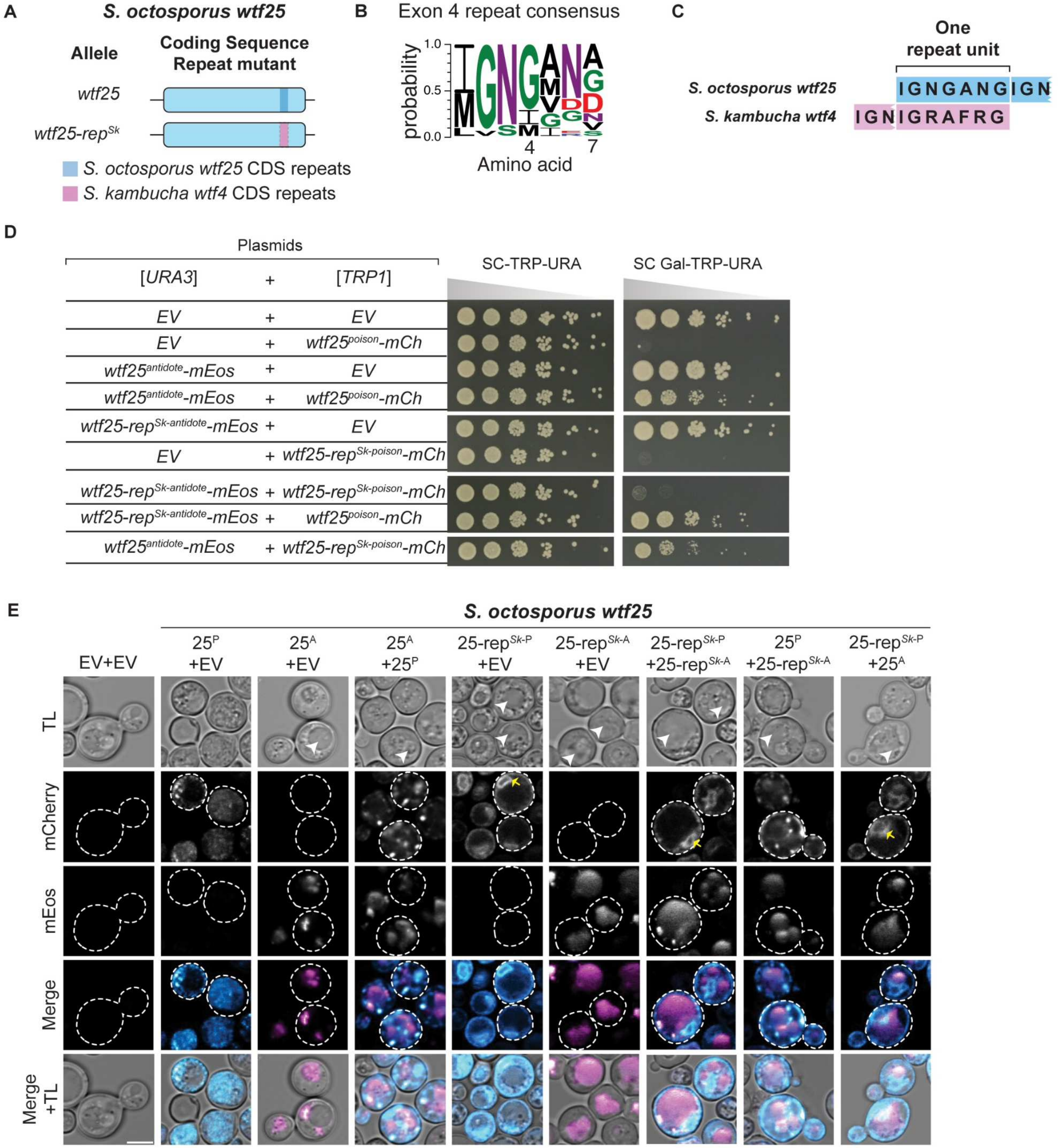
A
S. octosporus wtf25
Allele
Coding Sequence Repeat mutant
wtf25
wtf25-rep^Sk
S. octosporus wtf25 CDS repeats
S. kambucha wtf4 CDS repeats
B
Exon 4 repeat consensus
probability
1.0
0.5
0.0
4
7
Amino acid
C
One repeat unit
S. octosporus wtf25
IGNGANGIGN
S. kambucha wtf4
IGNIGRAFRG
D
Plasmids
[URA3] + [TRP1]
SC-TRP-URA
SC Gal-TRP-URA
EV + EV
EV + wtf25^poison-mCh
wtf25^antidote-mEos + EV
wtf25^antidote-mEos + wtf25^poison-mCh
wtf25-rep^Sk-antidote-mEos + EV
EV + wtf25-rep^Sk-poison-mCh
wtf25-rep^Sk-antidote-mEos + wtf25-rep^Sk-poison-mCh
wtf25-rep^Sk-antidote-mEos + wtf25^poison-mCh
wtf25^antidote-mEos + wtf25-rep^Sk-poison-mCh
E
S. octosporus wtf25
EV+EV
25^P +EV
25^A +EV
25^A +25^P
25-rep^Sk-P +EV
25-rep^Sk-A +EV
25-rep^Sk-P +25-rep^Sk-A
25^P +25-rep^Sk-A
25-rep^Sk-P +25^A
TL
mCherry
mEos
Merge
Merge +TL

S14 Figure

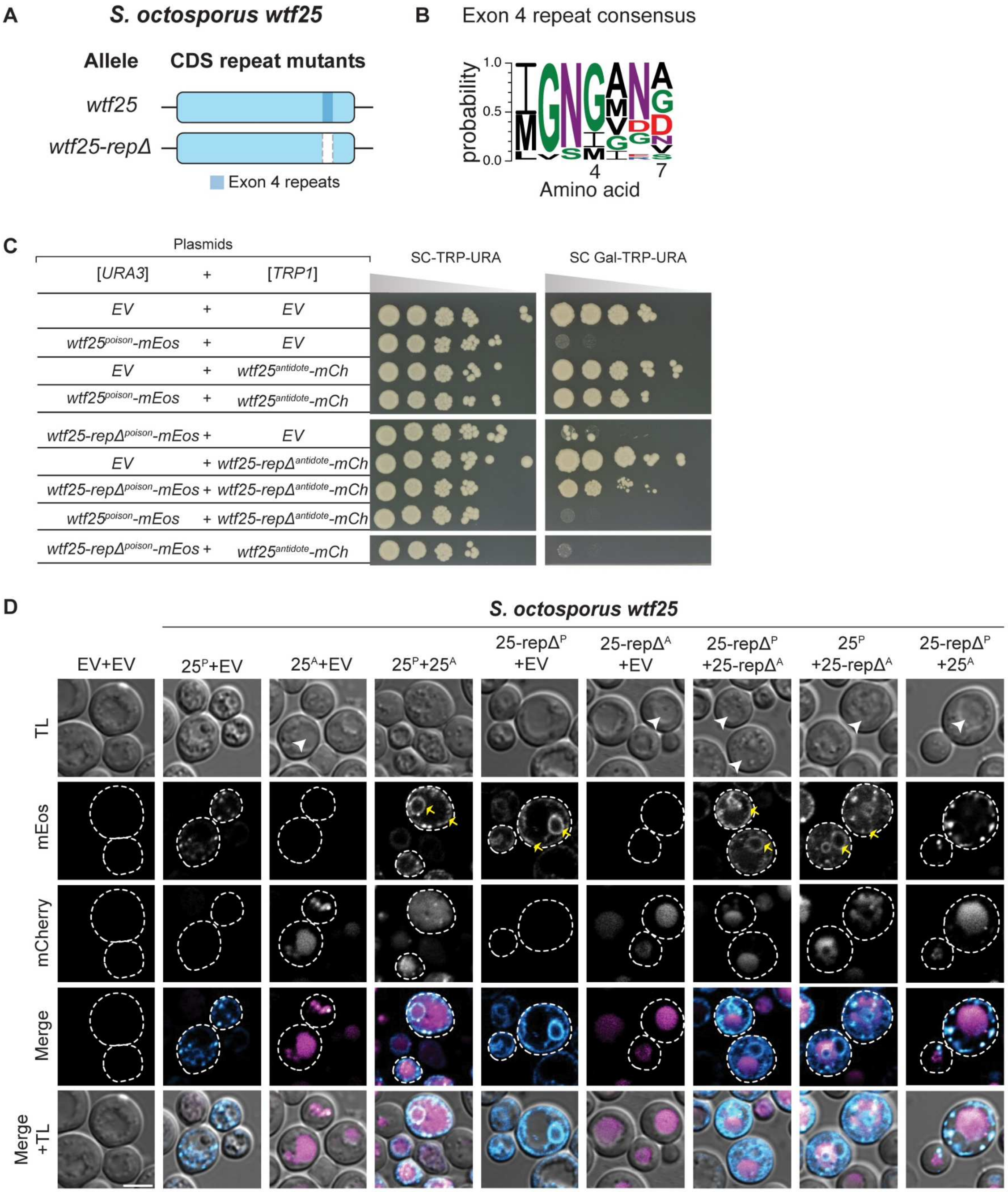
A
S. octosporus wtf25
Allele
CDS repeat mutants
wtf25
wtf25-repΔ
Exon 4 repeats
B
Exon 4 repeat consensus
probability
1.0
0.5
0.0
4
7
Amino acid
C
Plasmids
[URA3]
+
[TRP1]
SC-TRP-URA
SC Gal-TRP-URA
EV + EV
wtf25poison-mEos + EV
EV + wtf25antidote-mCh
wtf25poison-mEos + wtf25antidote-mCh
wtf25-repΔpoison-mEos + EV
EV + wtf25-repΔantidote-mCh
wtf25-repΔpoison-mEos + wtf25-repΔantidote-mCh
wtf25poison-mEos + wtf25-repΔantidote-mCh
wtf25-repΔpoison-mEos + wtf25antidote-mCh
D
S. octosporus wtf25
EV+EV
25P+EV
25A+EV
25P+25A
25-repΔP +EV
25-repΔA +EV
25-repΔP +25-repΔA
25P +25-repΔA
25-repΔP +25A
TL
mEos
mCherry
Merge
Merge +TL
